# FGFR2 is a Candidate Immune‐Associated Marker of Diabetic Foot Ulcer That Promotes Keratinocyte Function by Activating the PI3K/Akt and MAPK Pathways

**DOI:** 10.1155/mi/3260549

**Published:** 2026-04-15

**Authors:** Hailan Chen, Hongfei Sang, Yi Shi, Jie Pan, Chuang Zhang, Fengrui Lei

**Affiliations:** ^1^ Department of Vascular Surgery, The Second Affiliated Hospital of Soochow University, Suzhou, 215004, Jiangsu Province, China, suda.edu.cn

**Keywords:** diabetic foot ulcer, drug prediction, FGFR2, PI3K/Akt signaling

## Abstract

**Objective:**

Diabetic foot ulcer (DFU) is a serious complication of diabetes. This study aims to screen DFU–associated immune‐related genes (IRGs) and investigate their potential functional mechanisms and clinical relevance.

**Methods:**

Based on the GSE80178 dataset, the differentially expressed genes (DEGs) between DFU and diabetic foot skin (DFS) were screened out and cross‐linked with IRGs to obtain differentially expressed IRGs (DE‐IRGs). Functional enrichment analysis was conducted using the “clusterProfiler” R package. The protein–protein interaction (PPI) network was constructed using the STRING platform, and the core genes were identified based on topological analysis. The expression and diagnostic efficacy of core genes were verified using external datasets (GSE199939 and GSE134431). Immune cell infiltration analysis was performed using CIBERSORT algorithm. The candidate drugs were predicted through the L1000FWD database and molecular docking was carried out with Autodock Vina. The function of the core gene and its molecular mechanism were verified by constructing a high glucose (HG)–induced HaCaT cell injury model in vitro.

**Results:**

A total of 108 DE‐IRGs were screened out, including 48 upregulated genes and 60 downregulated genes. These genes were significantly enriched in the cytokine–cytokine receptor interaction, phosphoinositol 3‐kinase (PI3K)–protein kinase B (Akt) and mitogen‐activated protein kinase (MAPK) signaling pathways. Fibroblast growth factor receptor (FGFR) 2 was identified as the core gene, and its expression was significantly downregulated in DFU, with high diagnostic value (area under the curve [AUC] >0.95). The expression of FGFR2 was correlated with the infiltration levels of various immune cells. QL‐XI‐92, reversine, BRD‐K67414432, LY294002, and neratinib had high binding affinity with the FGFR2 protein. HG significantly reduced the expression of FGFR2 in HaCaT cells, inhibited proliferation and migration, and promoted apoptosis and the secretion of tumor necrosis factor‐α (TNF‐α), interleukin (IL)‐1β and IL‐6; overexpression of FGFR2 reversed the above‐mentioned phenomena and activated the PI3K/Akt and p38 MAPK pathways. The protective effect of FGFR2 could be reversed by LY294002 (an inhibitor of PI3K/Akt pathway) or SB202190 (a p38 MAPK inhibitor).

**Conclusion:**

FGFR2 is lowly expressed in DFU and can exert a protective effect by activating the PI3K/Akt pathway. It is a candidate diagnostic biomarker and potential therapeutic target for DFU.

## 1. Introduction

Diabetic foot ulcer (DFU) is one of the most serious chronic complications for diabetic patients. Its typical characteristics are persistent nonhealing wounds, often accompanied by infection, ischemia, and neuropathy, eventually leading to a high amputation rate and high mortality rate [1, 2]. Epidemiological investigations indicate that roughly 19%–34% of diabetic patients will progress to develop DFU, among which about 20% of the cases eventually require amputation surgery [3, 4]. Despite significant progress made in debridement techniques, wound dressings and anti‐infection treatments in recent years, the clinical therapeutic effect of DFU remains unsatisfactory, with a recurrence rate as high as 65% within 5 years [5].

In diabetic wounds, ischemia, hypoxia, and hyperglycemic microenvironments can disrupt programmed healing processes, resulting in the delayed healing or nonhealing of the wound surface and inducing diverse clinical complications [6]. Immune dysregulation assumes a pivotal role in the onset and progression of DFU [7–9]. Chronic inflammation can disrupt the balance of the immune system. Excessive pro‐inflammatory cytokines not only fail to promote tissue repair but also exacerbate tissue damage [10]. In addition, abnormal immune cell function, such as impaired transformation of macrophages from pro‐inflammatory phenotype to prohealing phenotype, leads to the continuous accumulation of inflammatory mediators, further hindering the healing of diabetic wounds [11, 12]. Therefore, clarification of the immune molecular mechanisms in DFU and the search for key immune‐related genes (IRGs) and their regulatory mechanisms are of certain scientific significance.

Fibroblast growth factor receptor (FGFR) 2 belongs to the receptor tyrosine kinase subfamily [13]. It activates downstream pathways by binding to fibroblast growth factors (FGFs), such as mitogen‐activated protein kinase (MAPK), phosphoinositol 3‐kinase (PI3K)/protein kinase B (Akt), phospholipase Cγ (PLCγ) pathways, and so on [14, 15]. FGFR2 is crucial for the proliferation and migration of keratinocytes and fibroblasts, and its downregulation impairs reepithelialization and granulation tissue formation, thereby resulting in persistent barrier defects [16, 17]. FGFR2 has been confirmed to be downregulated in human DFU tissues and diabetic mouse models [18]. However, its role in DFU development remains to be clarified.

This study aims to systematically identify and verify the role and mechanism of FGFR2, a core immune molecular marker in DFU, in the occurrence and development of DFU by integrating bioinformatics analysis and experimental verification. This work reports that FGFR2 is associated the dysfunction of immune cells in the microenvrionment of DFU, and overexpression of FGFR2 ameliorates the injury of keratinocyte induced by high glucose (HG) by regulating the PI3K/Akt and MAPK pathways.

## 2. Materials and Methods

### 2.1. Data Download and Processing

Using “diabetic foot ulcer” and “*Homo sapiens*” as key search terms, from Gene Expression Omnibus (GEO) database, DFU–related microarray datasets GSE80178, GSE199939, and GSE134431 were downloaded. The GSE80178 dataset (platform: GPL16686) contains three samples of diabetic foot skin (DFS) tissue and six samples of DFU tissue. GSE199939 (platform: GPL24676) contains 10 DFS tissue samples and 11 normal skin tissue samples. GSE134431 (Platform: GPL18573) contains 13 DFU tissue samples and eight DFS tissue samples. The data underwent background correction and normalization using the R package affy (version 1.80.0). The probe expression matrix was then converted into a gene expression matrix according to the respective platform annotation files, with missing values and probes matching multiple genes being removed. GSE80178 was used as the test dataset, and GSE199939 and GSE134431 were used as the validation datasets. Additionally, the “inSilicoMergeng” R package [19] was used to merge the DFU and DFS samples from the GSE80178 and GSE134431 datasets, creating a combined cohort as an additional validation set. Batch effect correction was performed using the ComBat function from the “sva” R package (version 3.52.0). “Immunity” was used as the search item, to obtain the IRGs from the Immport database (http://www.immport.org).

### 2.2. Screening and Functional Enrichment Analysis of Differentially Expressed IRGs (DE‐IRGs)

The “Limma” R package (version 3.66.0) was used to conduct differential expression analysis on the GSE80178 dataset to identify differentially expressed genes (DEGs) between DFU and DFS tissues (adjusted [adj.] *p*  < 0.05 and |log_2_ fold change [FC]| > 1). Volcano plots were generated for visualization using an online bioinformatics platform (http://www.bioinformatics.com.cn/). After cross‐analysis, the DE‐IRGs were obtained. Gene Ontology (GO) functional annotation and Kyoto Encyclopedia of Genes and Genomes (KEGG) pathway analysis of DE‐IRGs were performed using the “clusterProfiler” R package (version 4.18.4). An adj. *p*  < 0.05 was set as the significance threshold for enrichment. Based on the analysis results, enrichment bubble plots were generated for visualization using the bioinformatics online platform (http://www.bioinformatics.com.cn/).

### 2.3. Identification of Core Genes

The DE‐IRGs were imported into the STRING (version 12.0; https://string-db.org/) to construct a protein–protein interaction (PPI) network. After hiding disconnected nodes, the PPI network graph was obtained and saved in TSV format. Subsequently, the TSV file was opened with Cytoscape 3.9.0 software, and the top 15 genes ranked by algorithm scores including betweenness, closeness, degree, density of maximum neighborhood component (DMNC), maximal clique centrality (MCC), maximum neighborhood component (MNC), edge percolated component (EPC), radiality, and stress were retrieved using the CytoHubba plug‐in (version 3.5.2). The intersection of these genes was identified using the “UpSet” R package (version 1.4.0), and these overlapping genes were defined as the core genes.

### 2.4. Verification of Core Genes

Based on the GSE134431 dataset and the combined cohort (GSE80178 + GSE134431), the expression differences of core genes between the DFU and DFS groups were analyzed using the Wilcoxon rank–sum test. Receiver operating characteristic (ROC) curves were generated using the “pROC” R package (version 1.19.0.1) with single‐gene thresholding. The area under the curve (AUC) and its 95% confidence interval (CI) were calculated using the DeLong method. The optimal diagnostic threshold (cut‐off point) for each gene was determined by maximizing Youden’s index. Additionally, the expression differences of core genes between DFS and normal skin were analyzed using the GSE199939 dataset, following the same method as described above.

### 2.5. Assessment of Immune Cell Infiltration

To evaluate the immune microenvironment in DFU, immune cell infiltration analysis was performed based on the GSE80178 dataset using two deconvolution algorithms. First, the “CIBERSORT” R package (version 1.03) was applied to estimate the relative proportions of 22 immune cell subtypes in DFU and DFS tissues, and sample‑level *p*‑values were calculated to assess the confidence of the deconvolution results. Spearman correlation analysis was used to investigate the association between FGFR2 expression and the infiltration proportion of each immune cell subset. Multiple‐testing correction using the false discovery rate (FDR) method was applied to the differential infiltration results across the 22 immune subsets as well as to the correlation analyses between FGFR2 and immune cells. Given the limited sample size, the estimation of proportion of immune and cancer cells (EPIC) algorithm (version 1.1.7) was further employed as an orthogonal validation approach. EPIC was used to quantify the enrichment scores of key immune and stromal cells. Box plots were generated using the “ggplot2” R package (version 4.0.1) to visualize differences in infiltration between groups. In addition, Spearman correlation analysis was performed to examine the relationships between FGFR2 expression and the infiltration levels of key immune and stromal cells. Correlation results were visualized as lollipop plots using the bioinformatics online platform (http://www.bioinformatics.com.cn/). A *p*‑value <0.05 was considered statistically significant.

### 2.6. Drug Prediction and Molecular Docking Analysis

The DE‐IRGs related to DFU were divided into the upregulation group and the downregulation group, and imported into the L1000 Fireworks Display (L1000FWD) database (https://maayanlab.cloud/l1000fwd/) to obtain the top 10 drugs with the smallest *p*‐values in opposite signatures as candidate drugs. To evaluate the reliability of drug binding to the core protein FGFR2, molecular docking was conducted using AutoDock Vina v.1.1.2. The X‐ray crystal structure of FGFR2 protein was downloaded from the Protein Data Bank (PDB) database (https://www.rcsb.org/; PDB ID: 5EG3). The protein structure was dehydrated and hydrogenated using PyMOL software. From the PubChem (https://www.ncbi.nlm.nih.gov/pccompound/), the 2D chemical structure of candidate drugs were downloaded, and preserved in SDF format. Then, the SDF format of the drugs were converted to the PDB format with OpenBabel 3.1.1 software. Finally, the PDB files of proteins and drug molecules were converted to PDBQT format using AutoDockTools v1.5.7, and molecular docking was performed using AutoDock Vina. The results of molecular docking were visually displayed using PyMOL software.

### 2.7. Cell Culture

Human immortalized keratinocytes (HaCaT; CoBioer, Nanjing, China) and human umbilical vein endothelial cells (HUVECs; American Type Culture Collection, Manassas, VA, USA) were cultured in Dulbecco’s modified eagle’s medium (DMEM; Hyclone, Logan, UT, USA) supplemented with 10% fetal bovine serum (FBS) and 1% penicillin–streptomycin mixture (Hyclone), cultured in a constant temperature incubator at 37°C with 5% CO_2_. The medium was replaced every 2–3 days. HaCaT cells were stimulated with different concentrations of glucose (5.5, 10, 20, 30, 40, 50, and 60 mM D‐glucose; Sigma–Aldrich, Louis, MO, USA) for 24 and 48 h to determine the optimal conditions for constructing the in vitro DFU model. Normal glucose (NG; 5.5 mM D‐glucose) was used as the normal control. Mannitol (Sigma–Aldrich; 5.5 mM of D‐glucose + 24.5 mM of mannitol) was used as the osmotic control for HG.

The pcDNA3.1‐FGFR2 overexpression (FGFR2‐OE) plasmid, the pcDNA3.1 empty plasmid, small interfering RNA negative control (si‐NC), and three siRNAs targeting FGFR2 (si‐FGFR2#1, si‐FGFR2#2, and si‐FGFR2#3) were constructed and synthesized by GenePharma Co.,Ltd. (Shanghai, China). Lipofectamine 3000 transfection reagent (Thermo Fisher Scientific Inc., Waltham, MA, USA) was applied to transfect the plasmids or siRNAs into HaCaT cells and HUVECs. For pathway‐specific inhibition or activation experiments, the PI3K‐specific inhibitor LY294002 (10 µM; MedChem Express, Shanghai, China) or the p38 MAPK inhibitor SB202190 (10 µM; MedChem Express) was added upon switching to HG medium and maintained for 48 h. Correspondingly, the PI3K activator 740Y‐P (10 µM; MedChem Express) or the p38 MAPK activator anisomycin (10 µM; MedChem Express) were used in designated rescue studies. The cells in the control group were treated with an equal volume of dimethyl sulfoxide (DMSO; Sigma–Aldrich).

### 2.8. Real‐Time Quantitative Polymerase Chain Reaction (RT‐qPCR)

Total RNA was isolated from HaCaT cells via the utilization of TRIzol reagent (Invitrogen, Carlsbad, CA, USA). A total of 2 μg of the RNA was reverse‐transcribed into complementary DNA (cDNA) by employing the PrimeScript RT kit (Takara, Dalian, China). RT–qPCR was carried out in the CFX Connect real‐time PCR detection system (Bio–Rad, Hercules, CA, USA) with the use of 2 × SYBR Green RT‐qPCR Master Mix (Selleckchem, Houston, TX, USA). The primer sequences: FGFR2 forward 5′‐ACCGATGGTGCGGAAGATTT‐3′ and reverse 5′‐TCCAGTGCTGGTTTCGTACC‐3′; GAPDH forward 5′‐GGTCACCAGGGCTGCTTTTA‐3′ and reverse 5′‐CCCGTTCTCAGCCATGTAGT‐3′.

### 2.9. Western Blot

The total proteins of HaCaT cells were extracted using RIPA lysis buffer (Beyotime, Shanghai, China). Equal amounts of protein (20 μg) were separated by electrophoresis and transferred onto polyvinylidene fluoride membranes (Millipore, Billerica, MA, USA). After blocking with 5% skimmed milk at room temperature for 1 h, the membrane was linked to anti‐FGFR2 antibody (1:1000, ab289968, Abcam, Shanghai, China), anti‐PI3K antibody (1:1000, ab191606, Abcam), anti‐phospho (p)‐PI3K antibody (1:1000, ab278545, Abcam), anti‐AKT antibody (1:1000, ab179463, Abcam), anti‐p‐AKT antibody (1:1000, ab38449, Abcam), antimammalian target of rapamycin (mTOR) antibody (1:1000, ab134903, Abcam), anti‐p‐mTOR antibody (1:1000, ab131538, Abcam), anti‐p38 antibody (1:1000, ab170099, Abcam), anti‐p‐p38 antibody (1:1000, ab178867, Abcam), anti‐p‐FGFR2 antibody (1:1000, AF3285, R&D Systems, Inc., Minneapolis, MN, USA), anti‐FGFR substrate 2 (FRS2) antibody (1:1000, MAB4069, R&D Systems), anti‐p‐FRS2 antibody (1:1000, AF5126, R&D Systems), and anti‐GAPDH antibody (1:1000, ab181602, Abcam) at 4°C overnight. After washing three times with tris buffered saline tween (TBST), the membranes were incubated with the secondary antibody (1:5000, ab205718, Abcam) at room temperature for 2 h. The protein bands were detected using the BeyoECL Plus kit (Beyotime). Image acquisition and density value analysis were performed using ImageLab software version 4.1 (Bio‐Rad).

### 2.10. Cell Viability Assay

The HaCaT cells in each group were seeded in 96‐well plates (1 × 10^4^ cells, in 100 μL cell suspension per well), and cultured for 24 h. Then 10 μL of Cell Counting Kit‐8 (CCK‐8; Beyotime) solution was added into each well, and incubated in the incubator for 2 h. The absorbance (OD) value at a wavelength of 450 nm was detected using an microplate reader (Dynatech Labs, Chantilly, VA, USA).

### 2.11. Cell Migration Assay

HaCaT cells were resuspended in serum‐free medium (2 × 10^5^ cells/mL). Then, 100 μL of the cell suspension was added to the upper chamber of the Transwell chamber (8 μM pore size, Costar, Cambridge, MA, USA), and 600 μL of the complete medium was added to the lower chamber. The cells were incubated at 37°C for 24 h. Next, the cells passed through the pores were then fixed in 4% paraformaldehyde, air‐dried, and stained with 0.1% crystal violet. Finally, five fields of view (×200) were randomly selected under an inverted microscope, and the cells were counted.

### 2.12. Cell Apoptosis Assay

Apoptosis was detected using the annexin V‐fluorescein isothiocyanate (FITC)/propidium iodide (PI) apoptosis detection kit (Beyotime). The cells were washed twice with precooled PBS, centrifuged at 4°C and 1000 × *g* for 5 min, and the supernatant was discarded, and the cells were resuspended in 100 μL binding buffer, and gently mixed with the FITC and PI staining solution, and incubated at room temperature in the dark for 10 min. Next, 400 μL of binding buffer was added. Finally, the apoptosis was detected within 1 h using the FACScan flow cytometer (BD Biosciences, San Jose, CA, USA), and the apoptosis rate was evaluated by FlowJo V.10 software.

### 2.13. Enzyme‐Linked Immunosorbent Assay (ELISA)

The supernatants of cell culture medium was collected from each group, and centrifuged (4°C, 12000 × *g*) for 10 min to remove the cell debris. The concentrations of tumor necrosis factor‐α (TNF‐α), interleukin (IL)‐1β and IL‐6 in the cell supernatant were detected using the ELISA kit (Enzyme linked Biotechnology Co., Ltd., Shanghai, China).

### 2.14. Statistical Analysis

The data were expressed as “mean ± standard deviation (SD).” The data were statistically analyzed using SPSS 21.0 software (IBM Corp., Armonk, NY, USA). Intergroup comparisons were conducted using independent sample *t*‐tests or one‐way analysis of variance (ANOVA) and Tukey post hoc tests.  ^∗^ *p* < 0.05,  ^∗∗^ *p* < 0.01,  ^∗∗∗^ *p*  < 0.001. A *p* value <0.05 was considered statistically significant.

## 3. Results

### 3.1. Screening of DE‐IRGs

**Figure 1:**
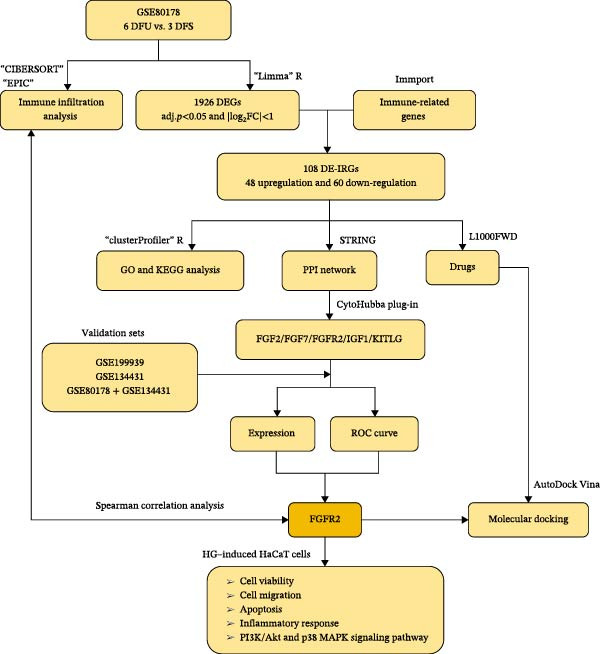
The workflow of this study. The schematic illustrates the integrative bioinformatics and experimental design. Bioinformatics discovery was performed using the GSE80178 dataset (platform: GPL16686; six DFU, and three DFS). Independent validation utilized GSE134431 (platform: GPL18573; 13 DFU, and eight DFS) and GSE199939 (platform: GPL24676; 10 DFS, and 11 normal skin). Core findings were functionally validated using an in vitro high glucose (HG)–induced HaCaT keratinocyte injury model.

The workflow of this study is shown in Figure 1. With the data in GSE80178, a total of 1926 significant DEGs were screened out, including 383 upregulated genes and 1543 downregulated genes (Figure 2A and Supporting Information 1: Table S1). Further cross‐analysis of DEGs and IRGs was conducted, resulting in 108 overlapping genes, namely, DE‐IRGs. Among them, 48 genes were upregulated and 60 genes were downregulated (Figure 2B and Supporting Information 1: Table S2). GO analysis showed that DE‐IRGs were enriched in a total of 728 biological processes (BPs), 10 cellular components (CCs), and 58 molecular functions (MFs). In BP, the DE‐IRGs were enriched in cell chemotaxis, myeloid leukocyte migration, leukocyte migration, leukocyte chemotaxis, and regulation of leukocyte migration (Figure 3A); in terms of CC, the DE‐IRGs were mainly associated with secretory granule lumen, cytoplasmic vesicle lumen, vesicle lumen, and collagen‐containing extracellular matrix, external side of plasma membrane (Figure 3A); as for MF, these genes were mainly involved in receptor ligand activity, signaling receptor activator activity, growth factor activity and cytokine activity, and cytokine receptor binding (Figure 3A). KEGG analysis indicated that DE‐IRGs were enriched in a total of 34 pathways. The top five pathways were cytokine–cytokine receptor interaction, viral protein interaction with cytokine and cytokine receptor, and PI3K–Akt signaling pathway, neuroactive ligand–receptor interaction, MAPK signaling pathway (Figure 3B).

Figure 2Screening of DE‐IRGs in DFU. (A) Volcano plot of DEGs between DFU (*n* = 6) and DFS (*n* = 3) tissues from the GSE80178 dataset. DEGs were identified using the limma R package with thresholds of adj. *p*  < 0.05 and |log_2_ FC| > 1. Yellow dots indicate upregulated genes, blue dots indicate downregulated genes, and gray dots indicate genes with insignificant differences. (B) Venn diagram showing the overlap between DEGs and IRGs to obtain DE‐IRGs.

**Figure:**
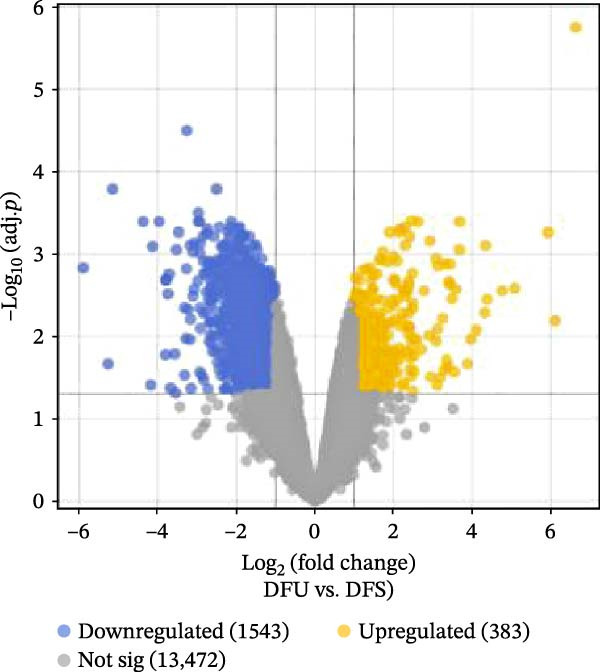
(A)

**Figure:**
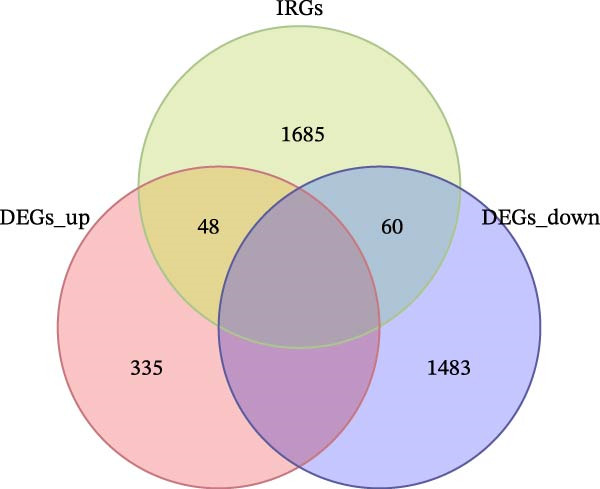
(B)

Figure 3Functional enrichment analysis of DE‐IRGs. (A) The bubble chart shows the results of GO analysis of DE‐IRGs (adj. *p*  < 0.05). BP, biological processes; CC, cellular components; MF, molecular function. (B) The bubble plot shows the results of KEGG pathway enrichment analysis of DE‐IRGs (adj. *p*  < 0.05). The size of the bubbles indicates the gene count. The color of the bubbles represents the adjusted *p* value.

**Figure:**
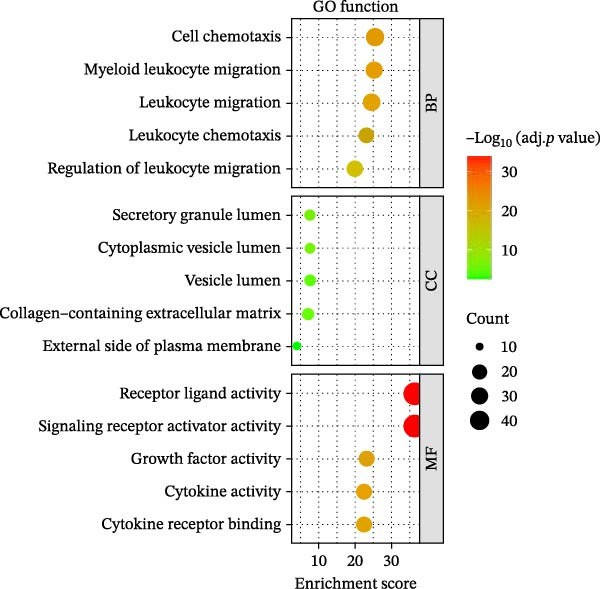
(A)

**Figure:**
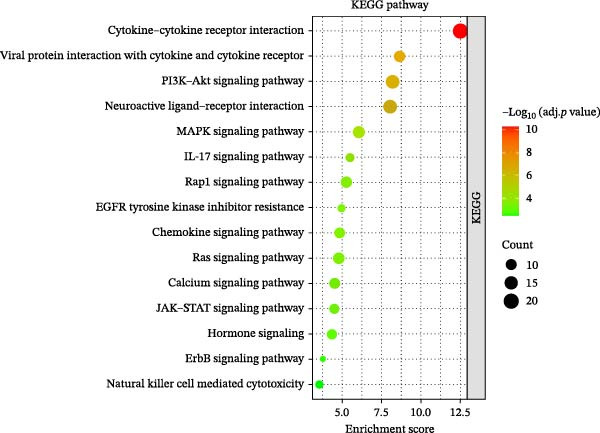
(B)

### 3.2. Identification and Verification of Core Genes

The PPI network of DE‐IRGs was constructed, consisting of 69 nodes and 131 edges (Figure 4A). Next, the top 15 genes with the highest scores of each algorithm were screened out in topological analyses (Table 1 and Supporting Information 1: Table S3). After further cross‐analysis, five overlapping genes were identified, namely, *FGF2*, *FGF7*, *FGFR2*, *IGF1*, and *KITLG* (Figure 4B), and are regarded as the potential crucial genes. To verify the expression level and diagnostic value of these core genes, we performed analyses on multiple validation sets. In GSE199939, the expression levels of FGF2, FGFR2, and IGF1 were decreased in the DFS group compared with normal skin tissue (Figure 4C). In GSE134431, the expression of FGFR2 in DFU samples was lower than that in DFS (Figure 4D). Furthermore, in the combined GSE80178 + GSE134431 cohort (DFU vs. DFS), FGFR2 expression was also downregulated in DFU (Figure 4E). ROC curves were then constructed to assess diagnostic performance. In GSE199939 (DFS vs. normal skin), FGFR2 achieved an AUC of 0.991 (95% CI: 0.962–1.000; Figure 4F). In GSE134431 (DFU vs. DFS), FGFR2 yielded an AUC of 0.981 (95% CI: 0.932–1.000; Figure 4G). Notably, in the combined GSE80178 + GSE134431 cohort (DFU vs. DFS), FGFR2 also exhibited outstanding diagnostic accuracy, with an AUC as high as 0.990 (95% CI: 0.966–1.000; Figure 4H). Together, these results confirmed that FGFR2 was consistently downregulated in DFU and had high diagnostic value for distinguishing DFU from DFS.

Figure 4Identification of core genes. (A) PPI network of the 108 DE‐IRGs constructed using STRING (confidence score = 0.7). The network consists of 69 nodes and 131 edges. (B) Intersection of top 15 genes from nine topological algorithms (betweenness, closeness, degree, DMNC, MCC, MNC, EPC, radiality, and stress) in CytoHubba, obtained using “UpSet” R package. (C) Box plots showing the expression differences of core genes (*FGF2*, *FGF7*, *FGFR2*, *IGF1*, and *KITLG*) between DFS (blue) and normal skin tissues (yellow) in the GSE199939 dataset, analyzed by Wilcoxon rank–sum test. (D, E) Box plots showing the expression differences of core genes (*FGF2*, *FGF7*, *FGFR2*, *IGF1*, and *KITLG*) between DFU (red) and DFS (blue) in the GSE134431 dataset (D) and the combined cohort (GSE80178 + GSE134431) (E), analyzed by Wilcoxon rank‐sum test. (F–H) ROC curves evaluating the diagnostic performance of core gene (*FGF2*, *FGF7*, *FGFR2*, *IGF1*, and *KITLG*) expression in the GSE199939 (F), GSE134431 (G), and combined cohort (GSE80178 + GSE134431) (H) datasets.

**Figure:**
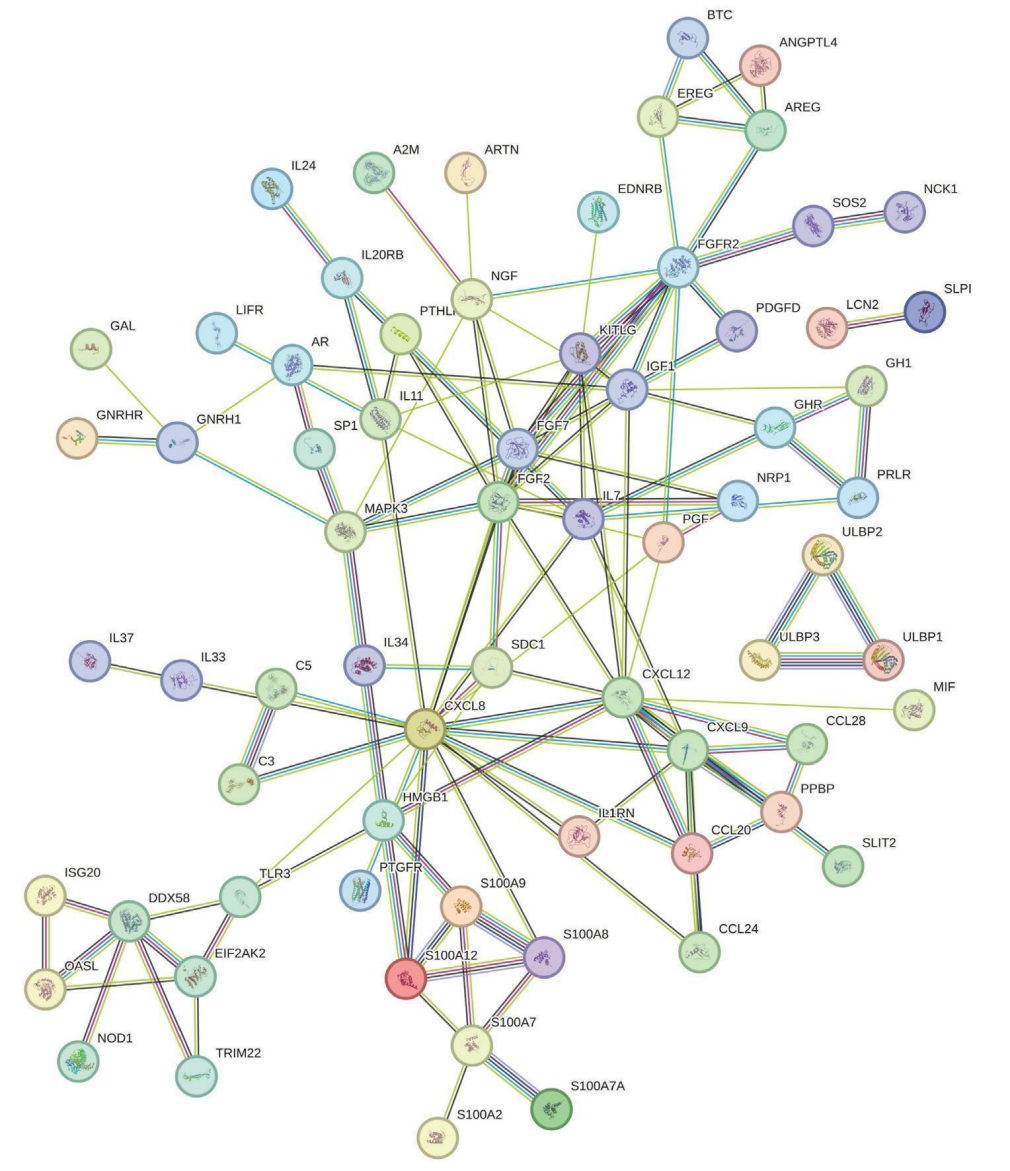
(A)

**Figure:**
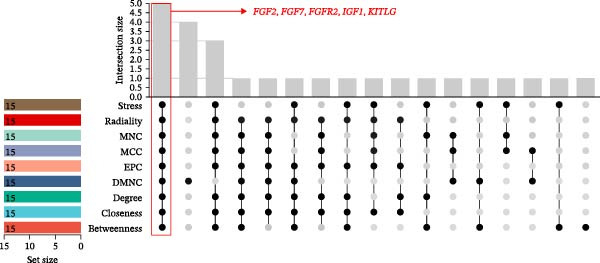
(B)

**Figure:**
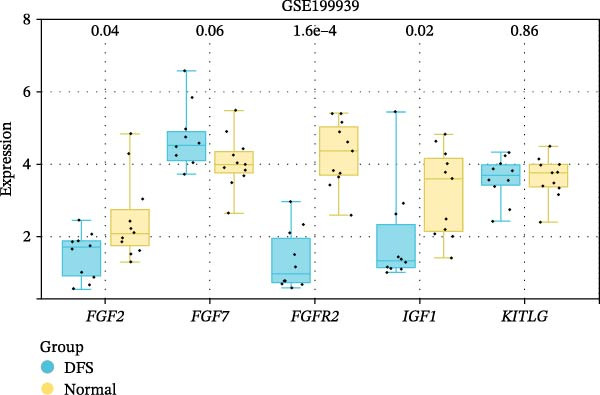
(C)

**Figure:**
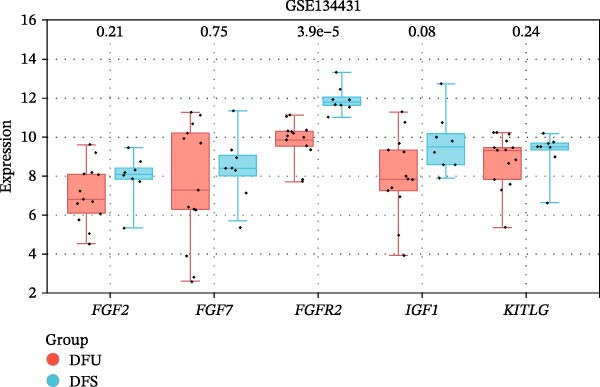
(D)

**Figure:**
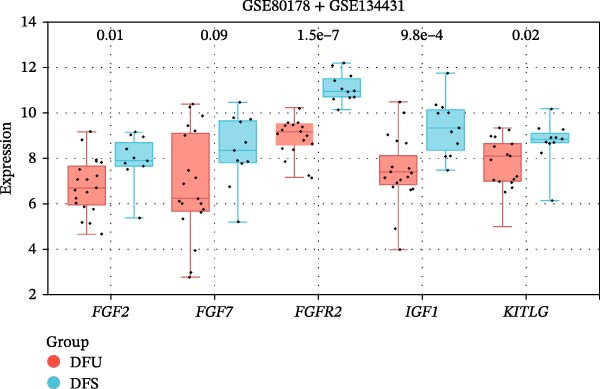
(E)

**Figure:**
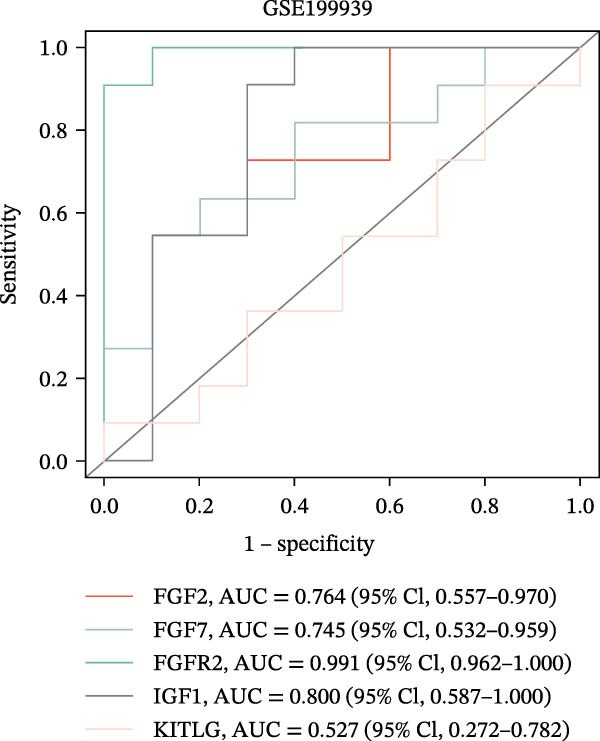
(F)

**Figure:**
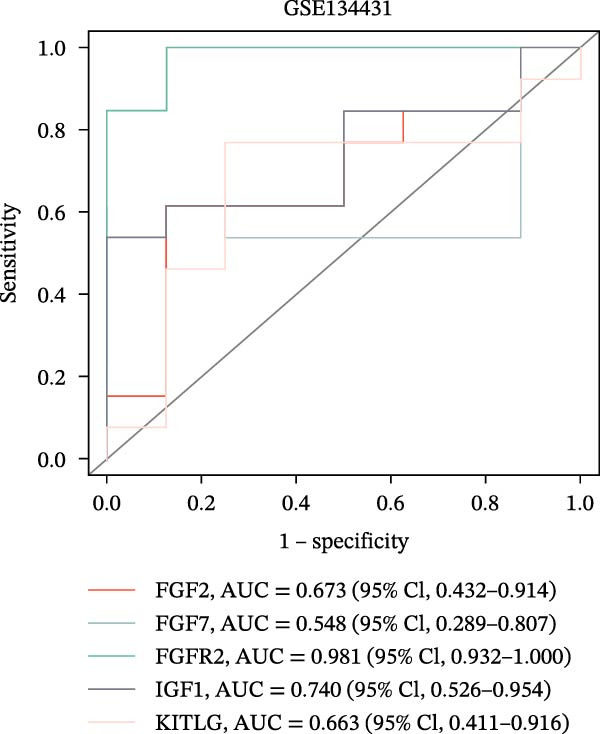
(G)

**Figure:**
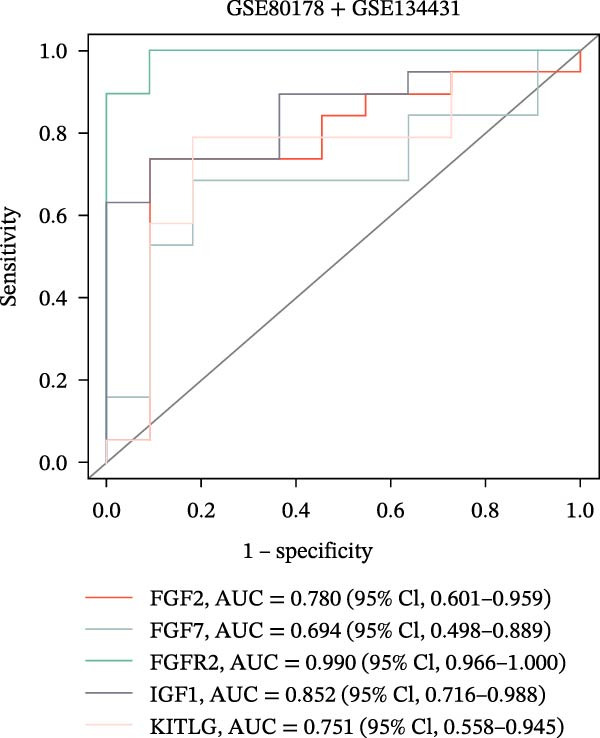
(H)

**Table 1:** The top 15 genes in terms of the scores of each algorithm in the CytoHubba plug‐in.

Betweenness	Closeness	Degree	DMNC	EPC	MCC	MNC	Radiality	Stress
CXCL8	CXCL8	CXCL8	NGF	FGF2	FGF2	FGF2	CXCL8	CXCL8
FGFR2	FGF2	CXCL12	PPBP	CXCL8	FGF7	CXCL8	FGF2	FGFR2
TLR3	CXCL12	FGF2	CCL24	CXCL12	IGF1	CXCL12	FGF7	FGF2
FGF2	FGF7	FGFR2	S100A7	FGF7	FGFR2	FGF7	CXCL12	TLR3
CXCL12	IGF1	IGF1	CCL28	IGF1	CXCL8	CXCL9	IL7	CXCL12
MAPK3	IL7	FGF7	MAPK3	KITLG	CXCL12	FGFR2	PGF	FGF7
IGF1	KITLG	IL7	CCL20	FGFR2	KITLG	KITLG	IGF1	IGF1
FGF7	FGFR2	CXCL9	IGF1	IL7	NGF	IGF1	SDC1	MAPK3
IL7	PGF	KITLG	FGFR2	CXCL9	CXCL9	IL7	KITLG	HMGB1
HMGB1	SDC1	NGF	SDC1	PGF	CCL20	CCL20	MAPK3	S100A7
DDX58	CXCL9	IL11	S100A8	SDC1	SDC1	S100A12	FGFR2	DDX58
NGF	NGF	DDX58	S100A9	NGF	IL7	NGF	IL11	IL7
KITLG	IL11	HMGB1	KITLG	HMGB1	PPBP	DDX58	HMGB1	KITLG
GNRH1	HMGB1	SDC1	FGF2	MAPK3	S100A12	SDC1	NGF	S100A12
S100A7	MAPK3	MAPK3	FGF7	IL11	PGF	PGF	CXCL9	PGF

Abbreviations: DMNC, density of maximum neighborhood component; EPC, edge percolated component; MCC, maximal clique centrality; MNC, maximum neighborhood component.

### 3.3. Analysis of Immune Infiltration

Inflammation is a key factor in the occurrence and development of DFU [20]. To assess the level of inflammation, the CIBERSORT algorithm was utilized to evaluate the infiltration of immune cells in the DFU. Sample‐level CIBERSORT p‐values were all >0.05 (Supporting Information 1: Table S4), indicating relatively low confidence of deconvolution results possibly due to small sample size. After FDR correction, none of the 22 immune cell subsets showed significant differential infiltration between DFU and DFS groups (all FDR > 0.05; Supporting Information 1: Table S5). Spearman correlation analysis indicated that the expression of FGFR2 was only negatively correlated with the infiltration level of natural killer (NK) cells resting after FDR correction (FDR = 0.001656; Supporting Information 1: Table S6), while no significant correlations were observed with other immune cells. We further performed immune infiltration analysis using the EPIC algorithm as an orthogonal validation. The results showed that compared with the DFS group, the infiltration levels of B cells (*p* = 0.02) and CD8^+^ T cells (*p* = 0.02) were increased in the DFU group, while the infiltration level of cancer‐associated fibroblasts (CAFs) was decreased (*p* = 0.02; Figure 5A). Spearman correlation analysis revealed that FGFR2 expression was negatively correlated with the infiltration levels of B cells (*r* = −0.82, *p* = 0.0072) and CD8^+^ T cells (*r* = −0.72, *p* = 0.0298; Figure 5B).

Figure 5Immune infiltration analysis results by EPIC algorithm. (A) Box plots showing the differential infiltration of key immune/stromal cells (B cells, CAFs, CD4^+^ T cells, CD8^+^ T cells, endothelial, macrophages, NK cells, other cells) between DFU (six samples, red) and DFS (three samples, blue) groups in GSE80178, analyzed by Wilcoxon rank‐sum test. (B) Spearman correlation lollipop plot between *FGFR2* expression and the infiltration levels of B cells, CAFs, CD4^+^ T cells, CD8^+^ T cells, endothelial, macrophages, NK cells, and other cells. The size of the point represents the absolute value of the correlation coefficient (*r*). The left side is negatively correlated, and the right side is positively correlated. The color represents the *p*‐value, and the bluer the color, the smaller the *p*‐value.

**Figure:**
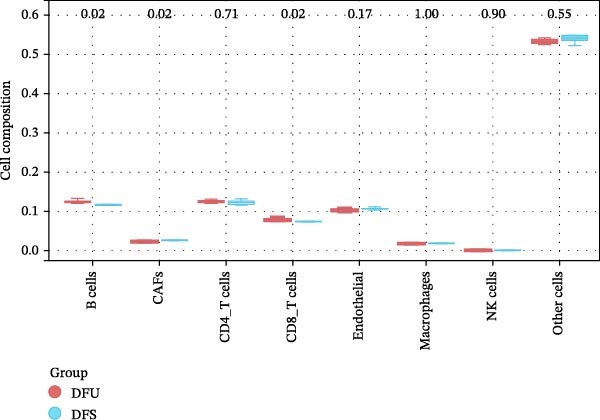
(A)

**Figure:**
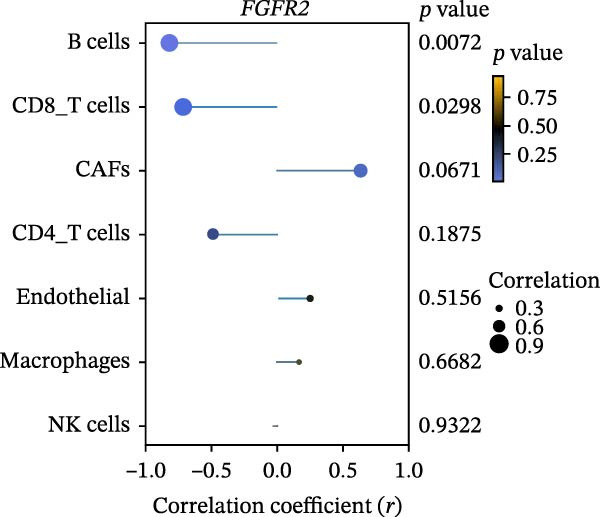
(B)

### 3.4. Drug Prediction and Molecular Docking Analysis

To explore potential therapeutic drugs for DFU, we conducted reverse screening of 108 DE‐IRGs using the L1000FWD database and obtained a total of 10 candidate drugs (Table 2). The binding affinities between QL‐XI‐92, duloxetine, PP‐1, BRD‐K69516039, reversine, lylamine, fludarabine, BRD‐K67414432, neratinib, LY294002, and FGFR2 protein were all ≤−6.5 kcal/mol, ranging from −9.9 to −6.9 kcal/mol (Table 2). Among them, QL‐XI‐92 (−9.9 kcal/mol), reversine (−9.2 kcal/mol), BRD‐K67414432 (−9.2 kcal/mol), LY294002 (−9.2 kcal/mol), and neratinib (−8.9 kcal/mol) exhibited the lowest binding energy, suggesting that their binding interaction with the FGFR2 protein was the most stable. Molecular docking showed that these five candidate drugs could stably bind with the amino acid residues of the FGFR2 protein through hydrogen bond interactions (Figure 6A–E). Specifically, QL‐XI‐92 primarily bound to residues GLY‐493, PHE‐492, and ALA‐491 (Figure 6A); reversine formed one hydrogen bond with ASP‐644 (Figure 6B); BRD‐K67414432 mainly interacted with ASP‐644 and GLU‐489, forming two hydrogen bonds (Figure 6C); LY294002 could engage with residue ALA‐567 (Figure 6D); neratinib primarily bound to residues LYS‐517 and GLU‐493 (Figure 6E).

Figure 6Molecular docking analysis. (A–E) Molecular docking diagrams of FGFR2 protein with QL‐XI‐92 (A), reversine (B), BRD‐K67414432 (C), LY294002 (D), and neratinib (E). Green represents small molecule drugs, light blue represent amino acid residues around the binding pocket, and the yellow dotted line indicates hydrogen bond forces.

**Figure:**
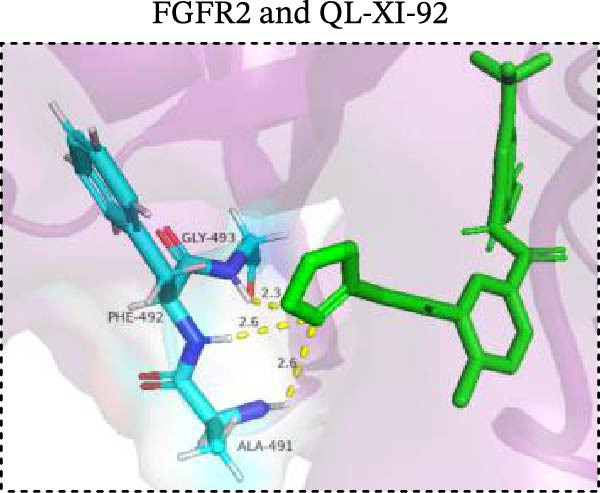
(A)

**Figure:**
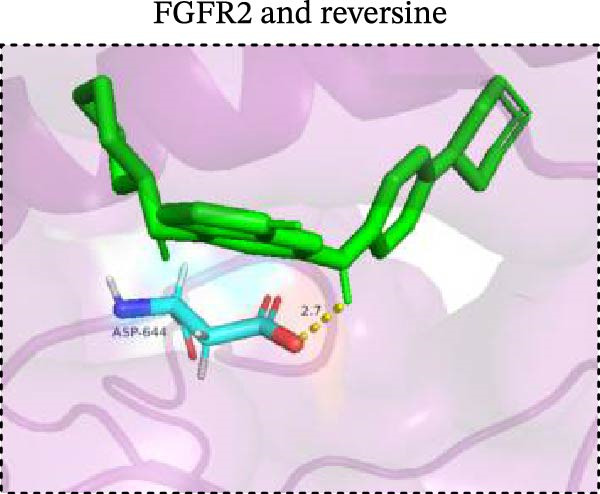
(B)

**Figure:**
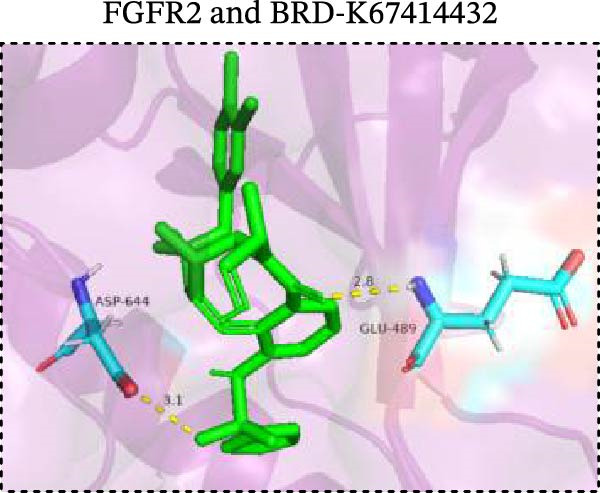
(C)

**Figure:**
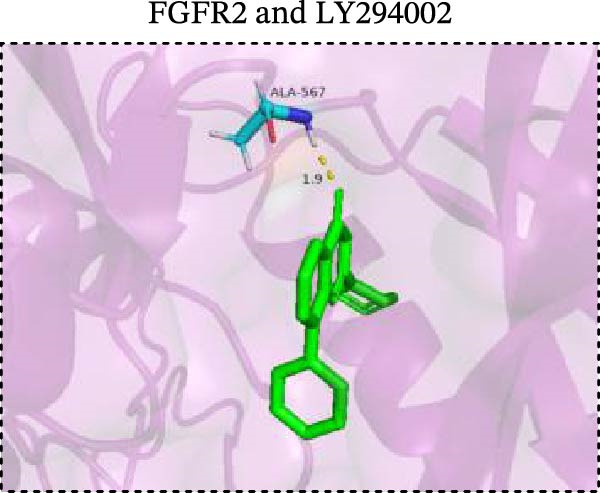
(D)

**Figure:**
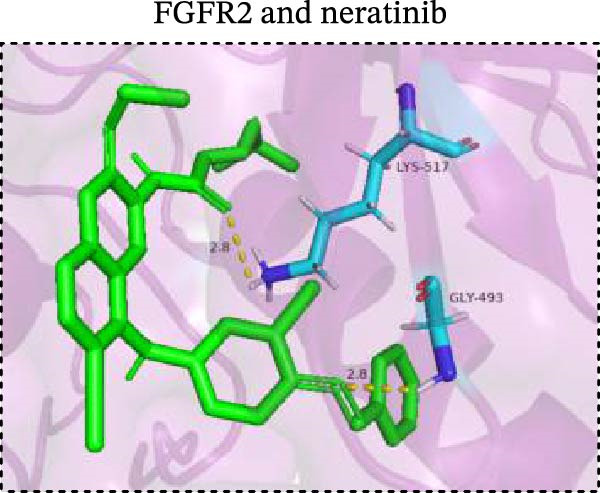
(E)

**Table 2:** Information on candidate drugs predicted by L1000FWD and statistics of molecular docking results.

Drug	Similarity score	*p*‐Value	Mechanism of action (MOA)	SMILES	Affinity (kcal/mol)	Chemical structure
QL‐XI‐92	−0.1596	3.32E−10	Unknown	Cc1ccc(cc1NC(═O)c1ccno1)C(═O)Nc1cc(NC(═O)C═C)cc(c1)C(F)(F)F	−9.9	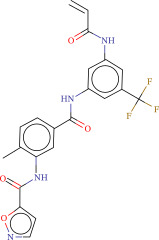
Duloxetine	−0.1383	4.09E−09	Norepinephrine reuptake inhibitor, serotonin–norepinephrine reuptake inhibitor (SNRI)	CNCC[C@H](Oc1cccc2ccccc12)c3cccs3	−8.1	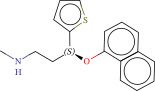
PP‐1	−0.1489	4.15E−09	src inhibitor	Cc1ccc(cc1)c2nn(c3ncnc(N)c23)C(C)(C)C	−8.6	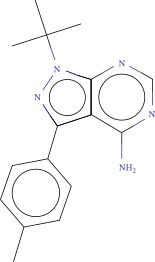
BRD‐K69516039	−0.1383	8.45E−09	Unknown	O═C(Nc1cc(ccc1N1CCCC1)S(═O)(═O)N1CCOCC1)c1cc2CCCCc2s1	−8.8	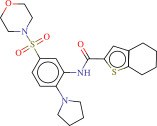
Reversine	−0.1383	1.16E−08	Aurora kinase inhibitor	C1CCC(CC1)Nc2nc(Nc3ccc(cc3)N4CCOCC4)nc5[nH]cnc25	−9.2	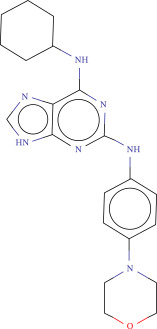
Lylamine	−0.1277	1.26E−08	Cannabinoid receptor agonist	CC(C)c1ccc2c(CC[C@H]3[C@](C)(CN)CCC[C@@]32C)c1	−8.7	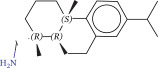
Fludarabine	−0.1383	1.94E−08	Ribonucleotide reductase inhibitor	Nc1nc(F)nc2n(cnc12)[C@@H]1O[C@H](CO)C(O)C1O	−6.9	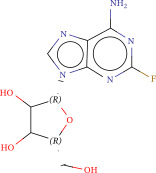
BRD‐K67414432	−0.1277	5.71E−08	Unknown	C[C@H](CO)N1C[C@H](C)[C@H](CN(C)Cc2ccc(Cl)c(Cl)c2)Oc3c(NS(═O)(═O)c4cccs4)cccc3C1═O	−9.2	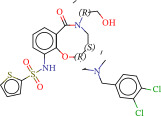
Neratinib	−0.1277	0.000000101	EGFR inhibitor	CCOc1cc2ncc(C#N)c(Nc3ccc(OCc4ccccn4)c(Cl)c3)c2cc1NC(═O)C═CCN(C)C	−8.9	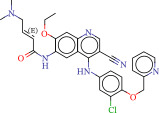
LY294002	−0.1277	0.000000122	mTOR inhibitor, PI3K inhibitor, DNA dependent protein kinase inhibitor, phosphodiesterase inhibitor, PLK inhibitor	O═c1cc(oc2c(cccc12)c3ccccc3)N4CCOCC4	−9.2	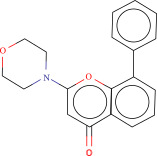

### 3.5. Overexpression of FGFR2 Alleviated HG–Induced Damage to HaCaT Cells

To determine the optimal glucose concentration for reducing HaCaT cell viability, the cells were treated with varying concentrations of glucose for 24 and 48 h. The results showed that glucose decreased HaCaT cell viability in a concentration‐ and time‐dependent manner, with IC_50_ values of 49.30 mM at 24 h and 33.27 mM at 48 h (Figure 7A). Therefore, a concentration of 30 mM glucose with a 48 h treatment was selected for subsequent experiments to establish the HG–induced injury model (Figure 7A). To exclude nonspecific osmotic effects, a mannitol treatment group was included as an osmotic control. The results indicated no significant difference in cell viability between the mannitol‐treated group and the NG group, demonstrating that the inhibitory effect of 30 mM glucose on HaCaT cell viability was not attributable to changes in osmotic pressure (Figure 7B). HG treatment reduced the mRNA and protein expression levels of FGFR2 in HaCaT cells (Figure 7C,D). The mRNA and protein expression levels of FGFR2 in HaCaT cells transfected with the FGFR2‐OE plasmid were increased (Figure 7E,F). Furthermore, in the context of HG, the expression levels of FGFR2 mRNA and protein in the HG + FGFR2‐OE group were higher than those in the HG + Vector group (Figure 7G,H), indicating that overexpression of FGFR2 could effectively reversed the inhibition of FGFR2 expression induced by HG. HG stimulation inhibited the viability and migration of HaCaT cells; however, overexpression of FGFR2 greatly reversed the inhibition of HaCaT cell viability and migration induced by HG (Figure 7I–K). Flow cytometry and ELISA assays showed that, the apoptosis level of HaCaT cells in the HG group was increased (Figure 7L,M), and the secretion of inflammatory cytokines (TNF‐α, IL‐1β, and IL‐6) was increased (Figure 7N–P). However, overexpression of FGFR2 could effectively weaken these effects (Figure 7I–P). The above results indicated that FGFR2 could play a protective role in HG–induced HaCaT cell injury by promoting cell proliferation and migration, inhibiting cell apoptosis and inflammatory response. To further verify the cell type universality of FGFR2’s protective effect, we performed functional validation in HG–induced HUVECs. The results showed that FGFR2 overexpression could also ameliorate HG–induced injury of HUVECs, with a protective effect consistent with that observed in HaCaT cells (Supporting Information 2: Figure S1.

Figure 7Effects of FGFR2 overexpression on HaCaT activity, migration, apoptosis, and inflammatory response induced by HG. (A) The CCK‐8 method was used to detect the viability of HaCaT cells treated with different concentrations of D‐glucose (5.5, 10, 20, 30, 40, 50, and 60 mM) for 24 or 48 h. (B) Cell viability of HaCaT cells cultured in 5.5 mM D‐glucose (NG), 30 mM D‐glucose (HG), or osmotic control mannitol (5.5 mM D‐glucose + 24.5 mM mannitol) for 48 h. (C, D) The mRNA and protein expression levels of FGFR2 in HaCaT cells induced by HG were detected by RT‐qPCR and western blot. (E, F) The expression levels of FGFR2 mRNA and protein in HaCaT cells transfected with FGFR2 overexpression plasmid and empty plasmid were detected by RT‐qPCR and Western blot. (G, H) The effects of FGFR2 overexpression on the expression levels of FGFR2 mRNA and protein in HG–induced HaCaT cells were detected by RT‐qPCR and western blot. (I–M) The effects of FGFR2 overexpression on the viability, migration (scale bar = 100 μm), and apoptosis of HaCaT cells induced by HG were detected by CCK‐8 assay (I), transwell assay (J, K), and flow cytometry (L, M), respectively. (N–P) The effect of FGFR2 overexpression on the levels of TNF‐α (N), IL‐1β (O), and IL‐6 (P) in HG–induced HaCaT cells was detected by ELISA. The data are expressed as mean ± SD, with *n* = 3 independent biological replicates.  ^∗∗∗^ represents *p*  < 0.001.

**Figure:**
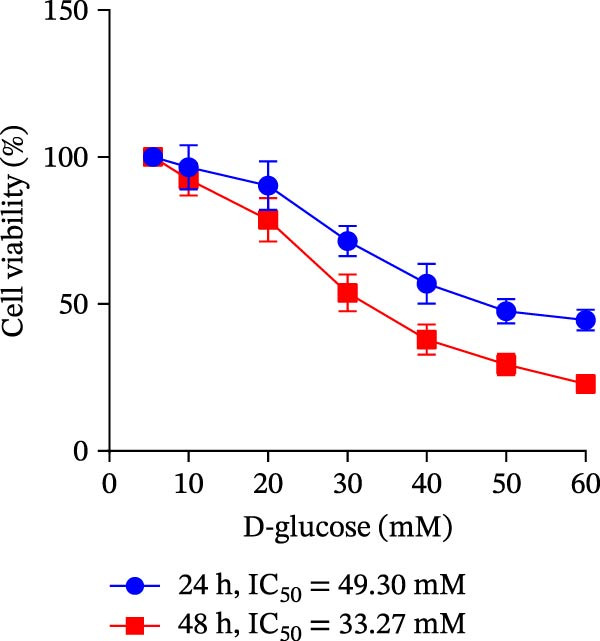
(A)

**Figure:**
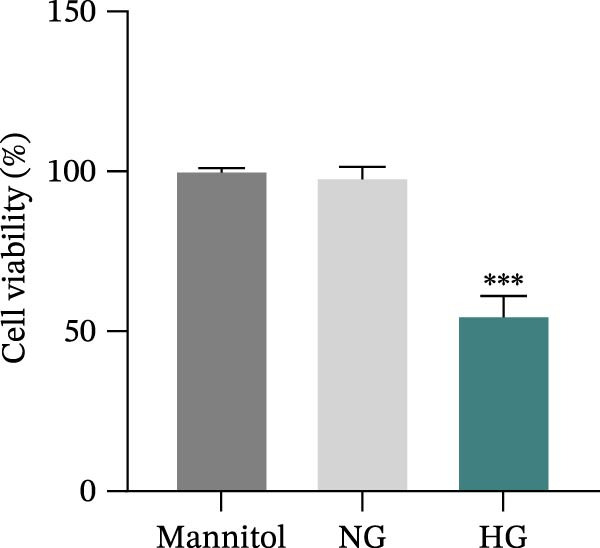
(B)

**Figure:**
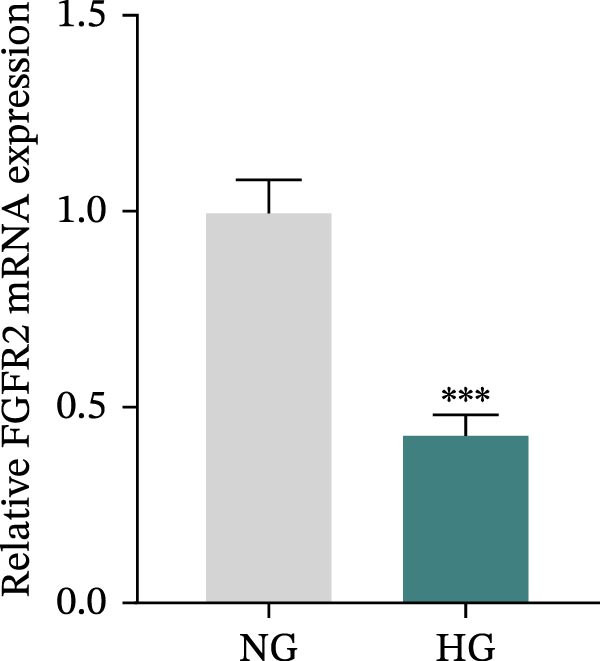
(C)

**Figure:**
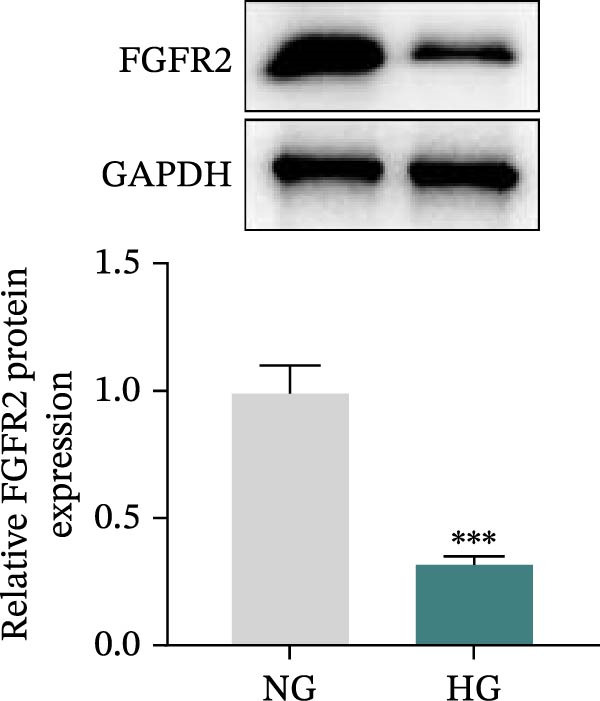
(D)

**Figure:**
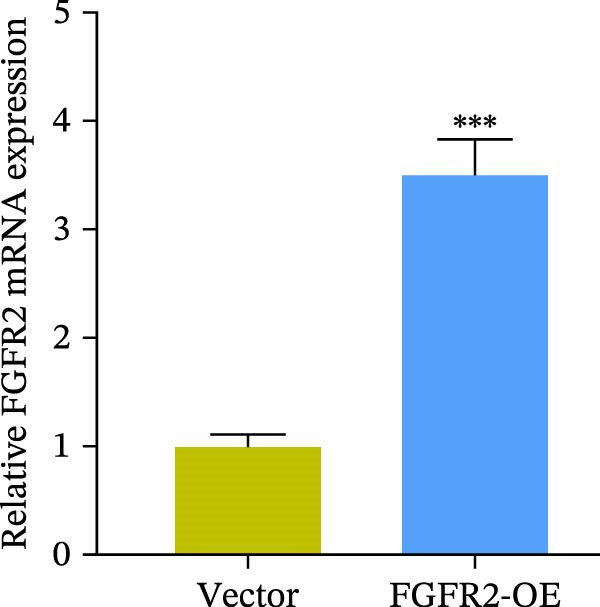
(E)

**Figure:**
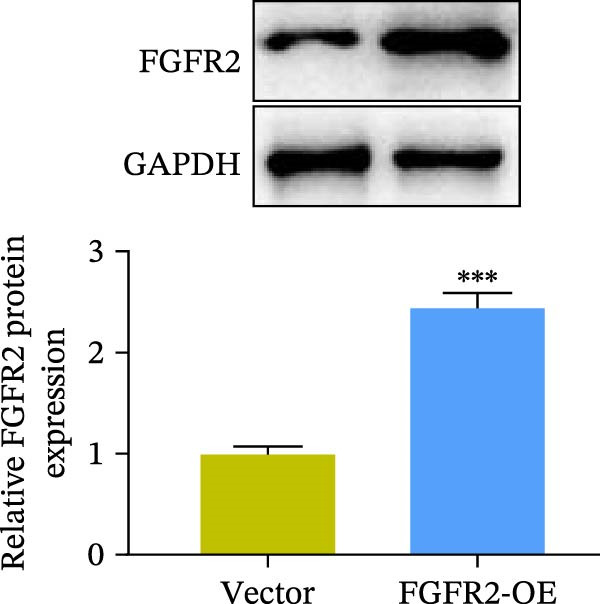
(F)

**Figure:**
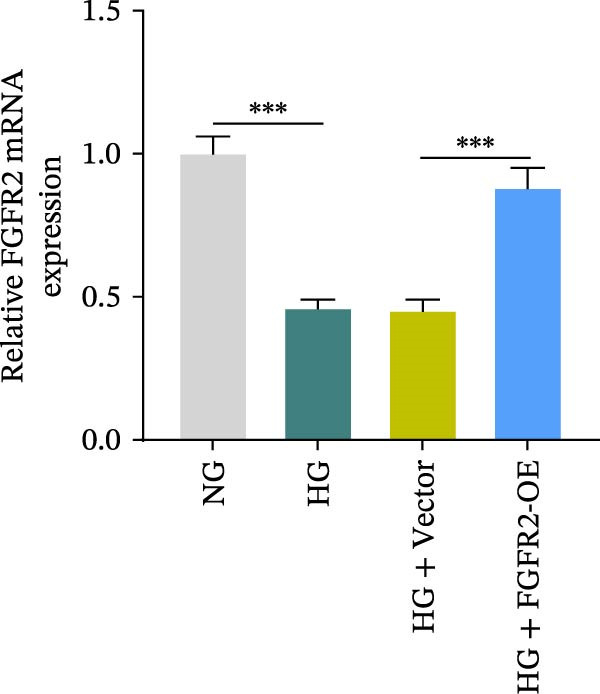
(G)

**Figure:**
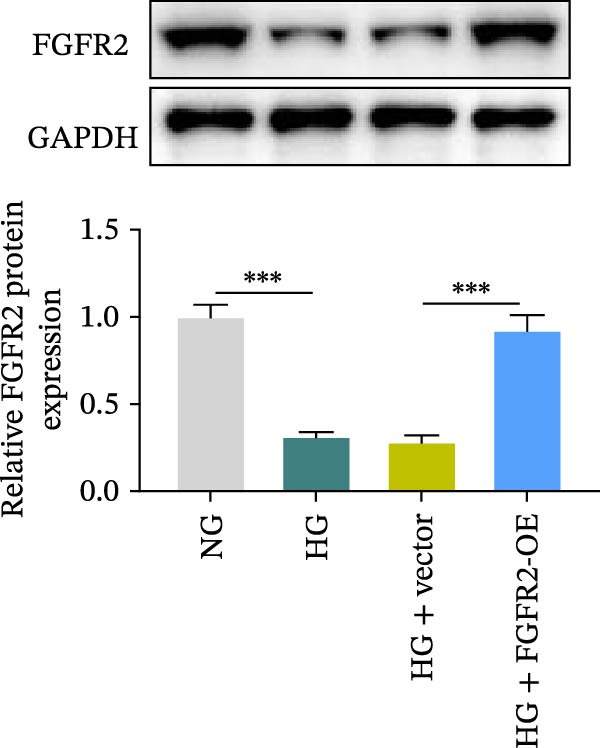
(H)

**Figure:**
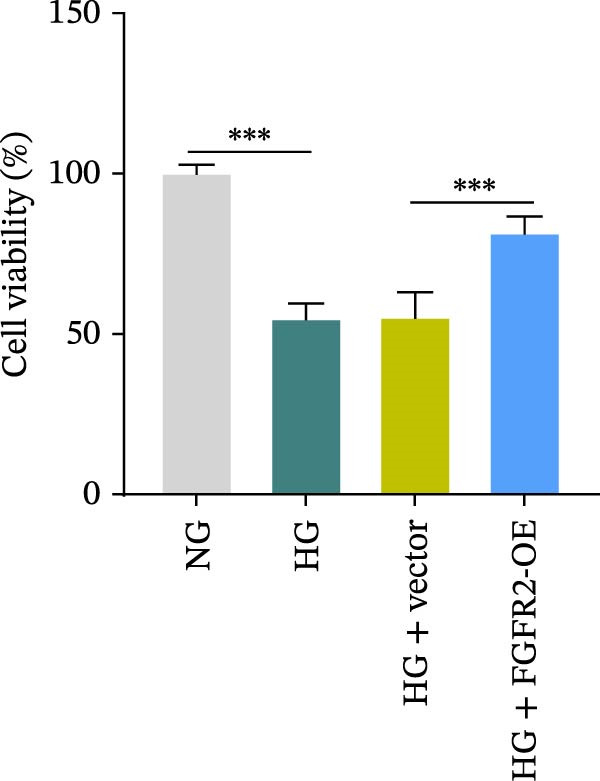
(I)

**Figure:**
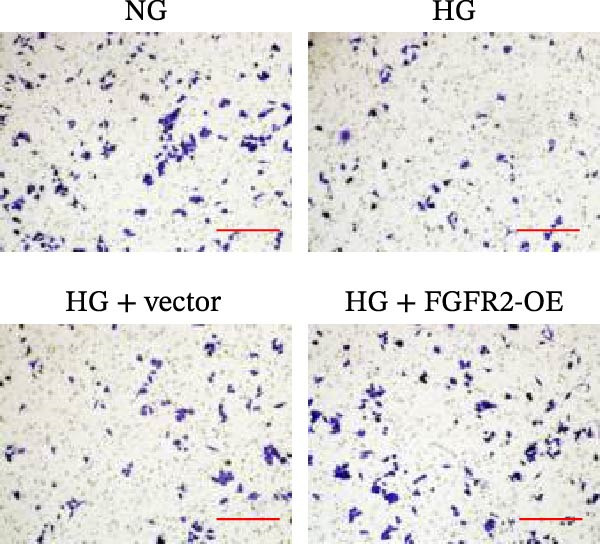
(J)

**Figure:**
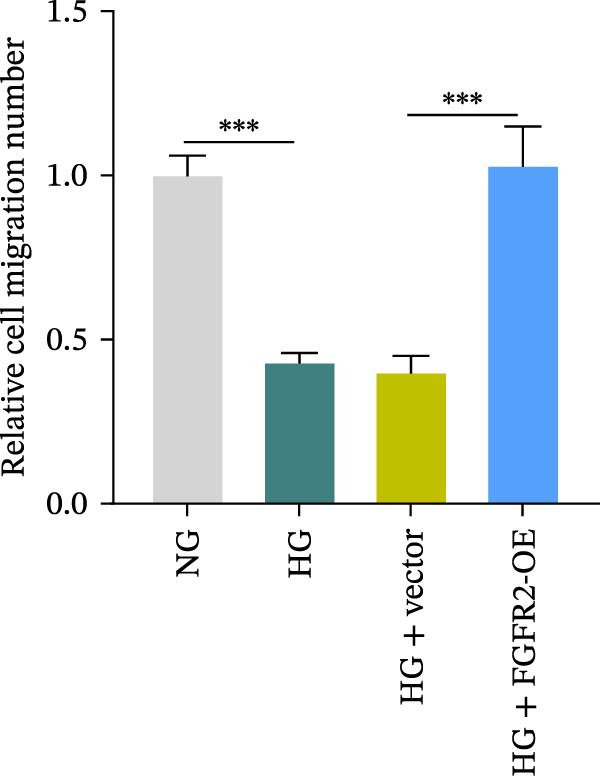
(K)

**Figure:**
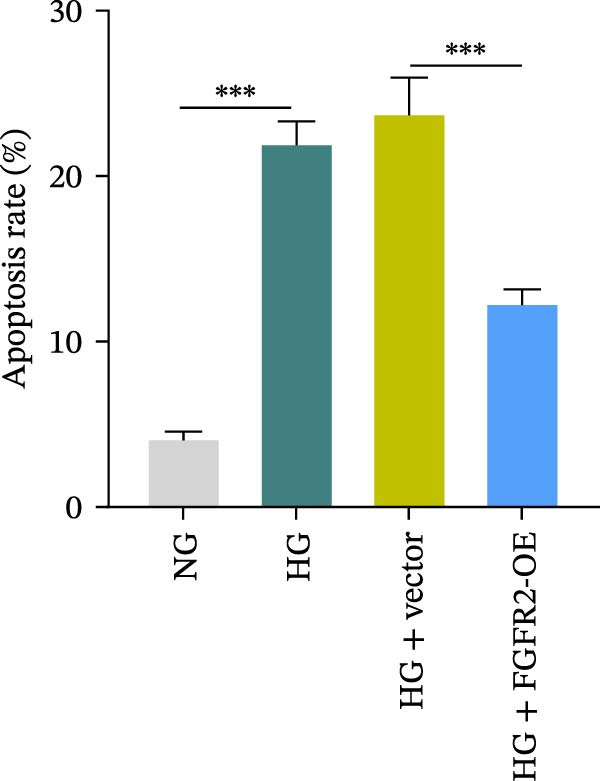
(L)

**Figure:**
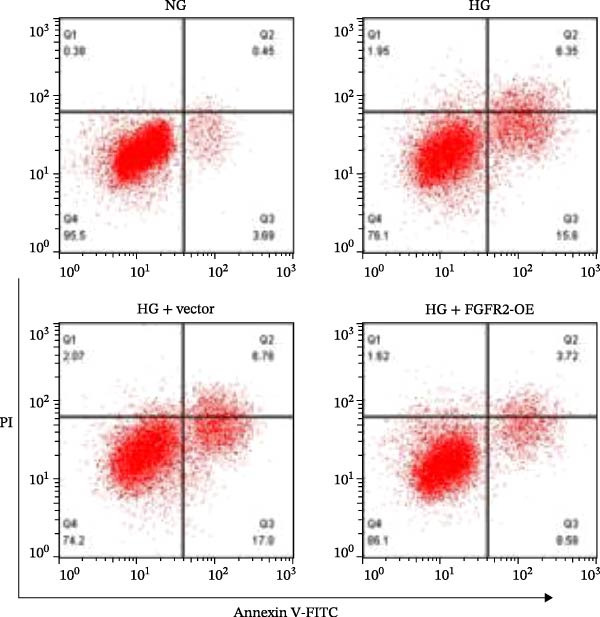
(M)

**Figure:**
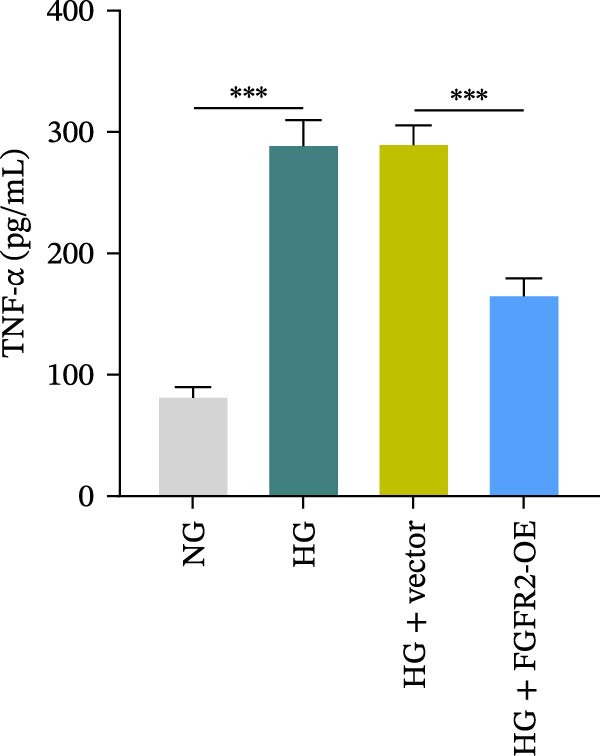
(N)

**Figure:**
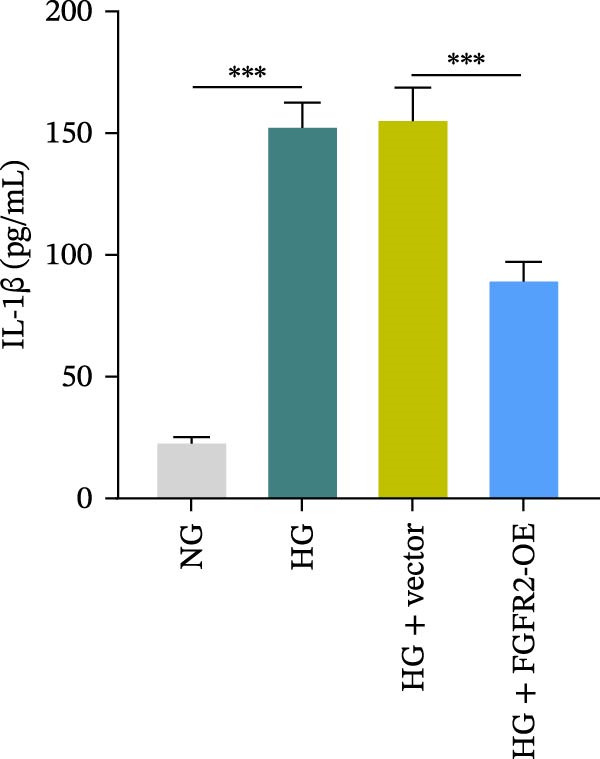
(O)

**Figure:**
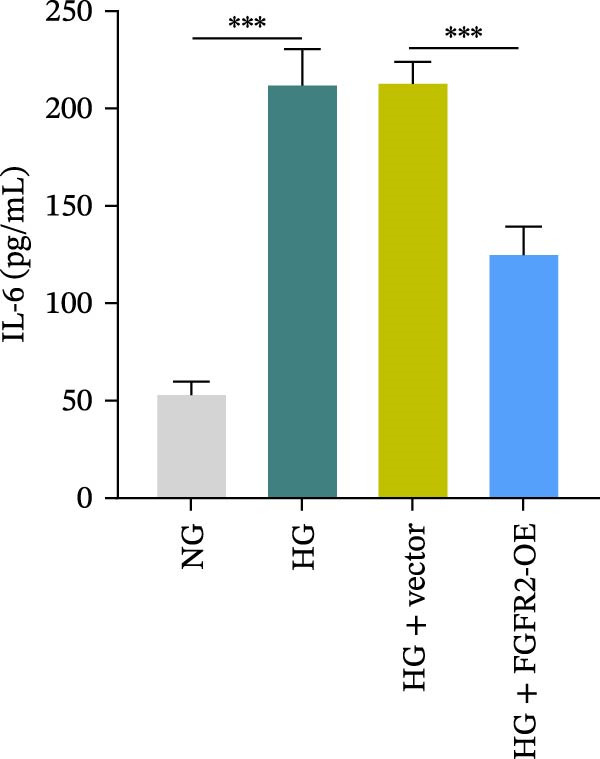
(P)

### 3.6. Overexpression of FGFR2 Activates Both PI3K/Akt and p38 MAPK Pathways in HG–Induced HaCaT Cells

Next, we constructed a PPI network of FGFR2 and screened out 50 proteins that interacted with the FGFR2 protein. GO analysis showed that FGFR2‐related targets were enriched in the FGFR signaling pathway (Figure 8A). The KEGG pathway analysis results show that FGFR2‐related targets were enriched in multiple pathways such as Ras signaling pathway, Rap1 signaling pathway, PI3K–Akt signaling pathway, and MAPK signaling pathway (Figure 8B). Interestingly, the results of western blot showed that compared with the NG group, HG stimulation downregulated the expressions of p‐PI3K, p‐AKT, p‐mTOR, and p‐p38 in HaCaT cells; the overexpression of FGFR2 promoted the expression of p‐PI3K, p‐AKT, p‐mTOR, and p‐p38 in HG–induced HaCaT cells (Figure 8C,D). To delineate the downstream signaling cascades of FGFR2 more precisely, we examined the levels of p‐FGFR2 and p‐FRS2. Western blot revealed that overexpression of FGFR2 reversed the HG–induced reduction in p‐FGFR2 and p‐FRS2 levels (Figure 8D). This suggested that FGFR2 activated the PI3K/Akt and p38 MAPK pathways in HG–induced HaCaT cells.

Figure 8Functional enrichment analysis of *FGFR2*–related targets. (A, B) The bubble plots show the results of GO functional annotation of FGFR2‐related targets (A) and KEGG pathway enrichment analysis (B). The size of the bubbles indicates the gene count. The color of the bubbles represents the adjusted *p* value. (C, D) Western blot was used to detect the effects of *FGFR2* overexpression on the expression levels of p‐PI3K, p‐AKT, p‐mTOR, p‐p38, p‐FGFR2, and p‐FRS2 in HG–induced HaCaT cells. The data is expressed as mean ± SD, with *n* = 3 independent biological replicates.  ^∗∗∗^ represents *p*  < 0.001.

**Figure:**
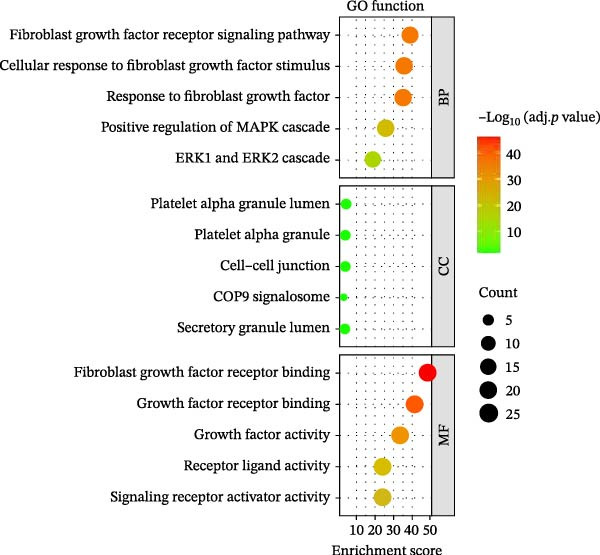
(A)

**Figure:**
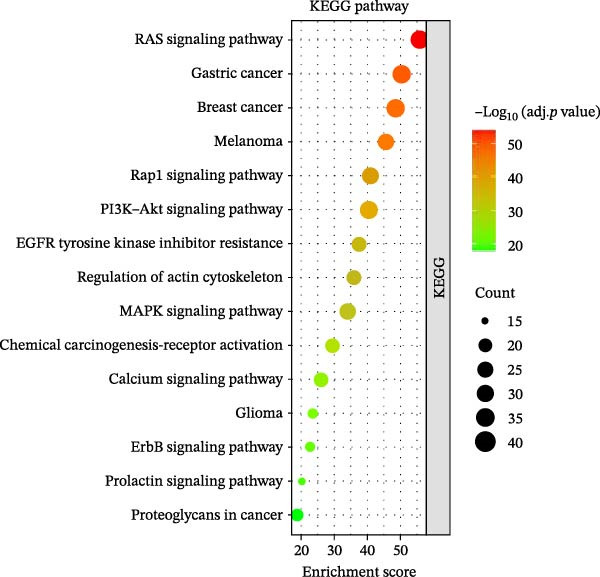
(B)

**Figure:**
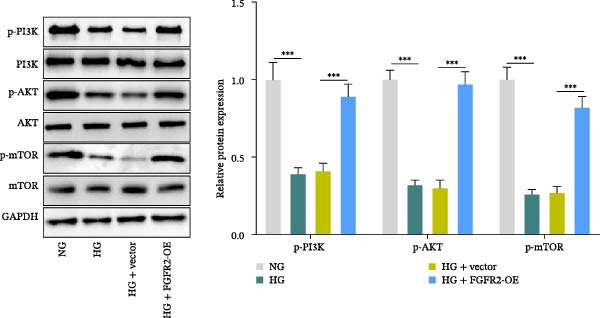
(C)

**Figure:**
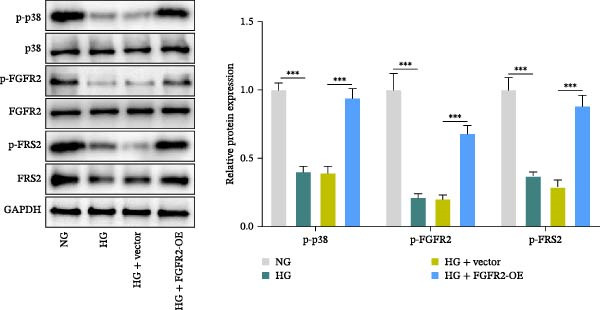
(D)

### 3.7. PI3K and p38 MAPK Inhibitors Reversed the Protective Effect of FGFR2 Overexpression on HG–Induced HaCaT Cells

To verify whether FGFR2 regulates HG–induced functional impairment and inflammatory response in HaCaT cells through the PI3K/Akt or p38 MAPK pathways, the PI3K inhibitor LY294002 and the p38 MAPK inhibitor SB202190 were applied. Overexpression of FGFR2 increased the expression levels of p‐PI3K, p‐AKT, p‐mTOR, and p‐p38 proteins in HG–induced HaCaT cells (Figure 9A–C). These increases were attenuated by treatment with either LY294002 or SB202190 (Figure 9A–C). In addition, LY294002 or SB202190 weakened the promoting effect of FGFR2 overexpression on HG–induced viability and migration of HaCaT cells (Figure 9D,E). Also, overexpression of FGFR2 inhibited HG–induced apoptosis of HaCaT cells, while LY294002 or SB202190 treatment reversed this phenomenon (Figures 9F). ELISA showed that overexpression of FGFR2 inhibited the levels of TNF‐α, IL‐1β, and IL‐6 in HG–induced HaCaT cells; however, both LY294002 and SB202190 partially weakened the inhibitory effect of FGFR2 overexpression on the levels of the abovementioned inflammatory factors (Figure 9G–I). Furthermore, western blot confirmed that the enhancing effect of FGFR2 overexpression on the levels of p‐FGFR2 and p‐FRS2 in HG–induced HaCaT cells was also diminished by LY294002 or SB202190 (Figure 9J). These results suggested that the protective effect of FGFR2 on HG–induced HaCaT cell injury was achieved through the concerted activation of both the PI3K/Akt and p38 MAPK signaling pathways.

Figure 9Both PI3K and p38 MAPK inhibitors reversed the protective effect of FGFR2 overexpression on HG–induced HaCaT cells. (A–C) Protein expression levels of p‐PI3K, p‐Akt, p‐mTOR, and p‐p38 in HG–induced HaCaT cells transfected with FGFR2‐OE and treated with LY294002 or SB202190, detected by western blot. (D) Cell viability of HG–induced HaCaT cells with FGFR2 overexpression and LY294002/SB202190 treatment, detected by CCK‐8 assay. (E) Cell migration ability of HG–induced HaCaT cells with FGFR2 overexpression and LY294002/SB202190 treatment, evaluated by transwell assay. Scale bar = 100 μm. (F) Apoptosis of HG–induced HaCaT cells with FGFR2 overexpression and LY294002/SB202190 treatment, analyzed by flow cytometry. (G–I) The levels of TNF‐α (G), IL‐1β (H), and IL‐6 (I) in HG–induced HaCaT cells with FGFR2 overexpression and LY294002/SB202190 treatment, detected by ELISA. (J) Protein expression levels of p‐FGFR2 and p‐FRS2 in HG–induced HaCaT cells transfected with FGFR2‐OE and treated with LY294002 or SB202190, detected by western blot. The data is expressed as mean ± SD, with *n* = 3 independent biological replicates.  ^∗∗^ and  ^∗∗∗^, respectively, represent *p*  < 0.01 and *p*  < 0.001.

**Figure:**
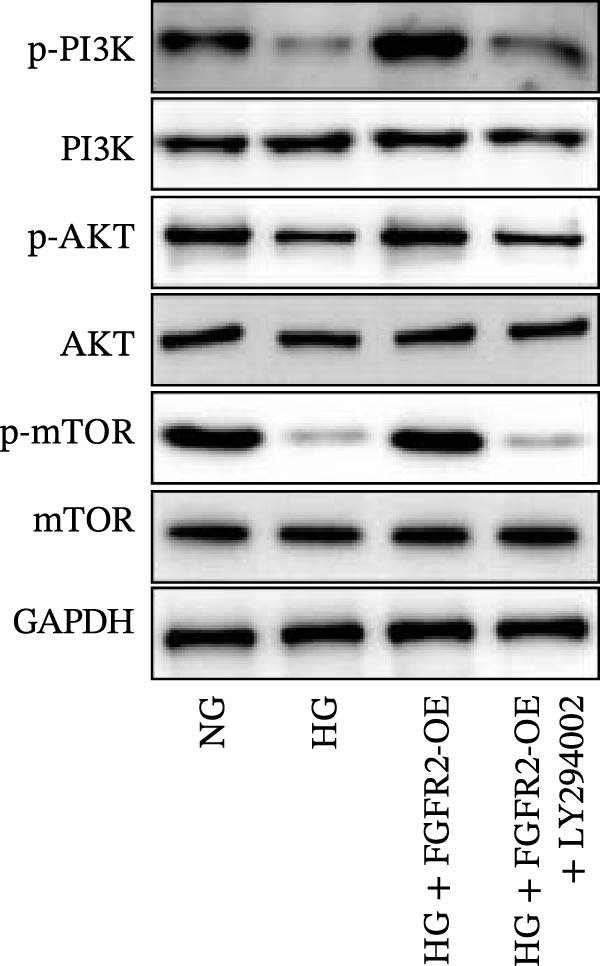
(A)

**Figure:**
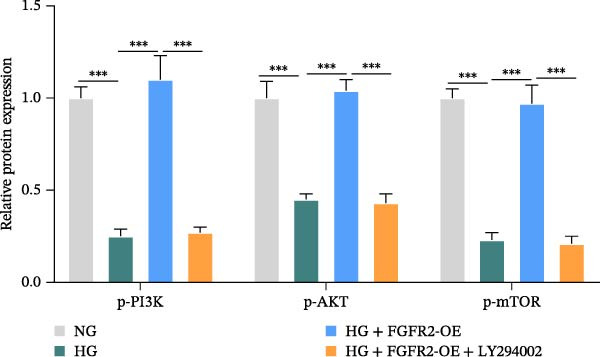
(B)

**Figure:**
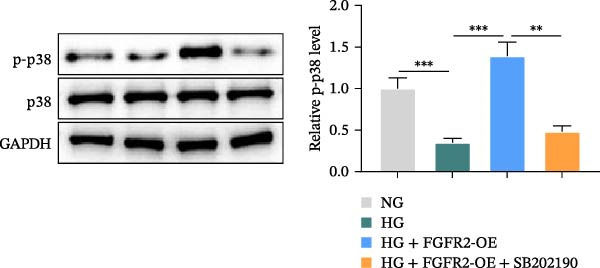
(C)

**Figure:**
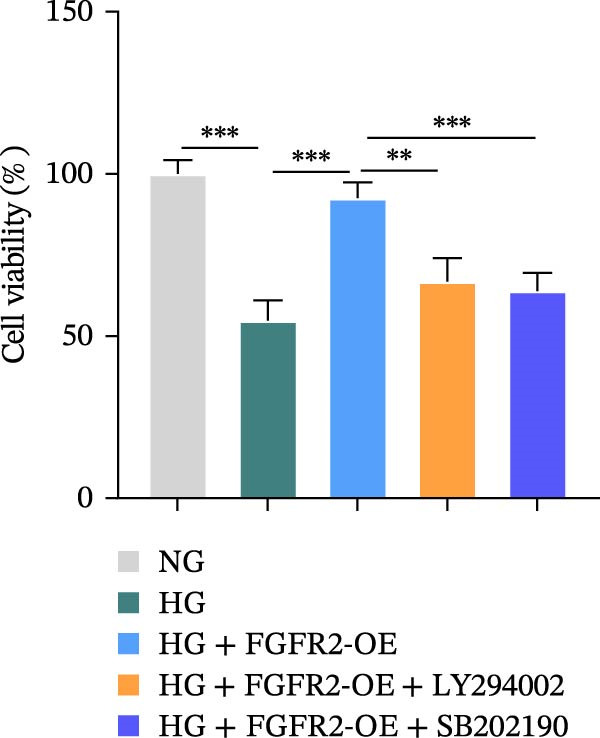
(D)

**Figure:**
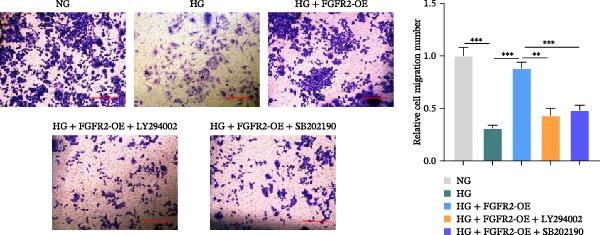
(E)

**Figure:**
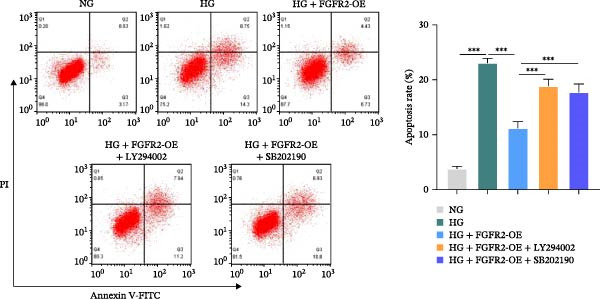
(F)

**Figure:**
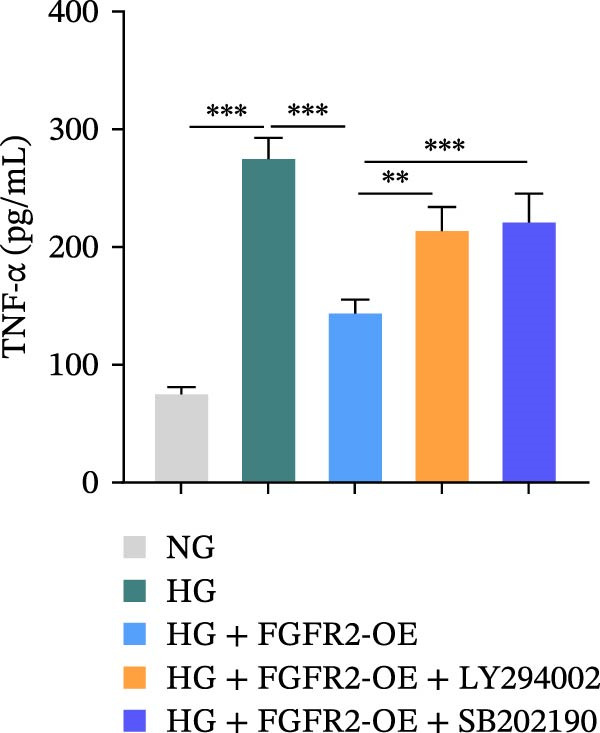
(G)

**Figure:**
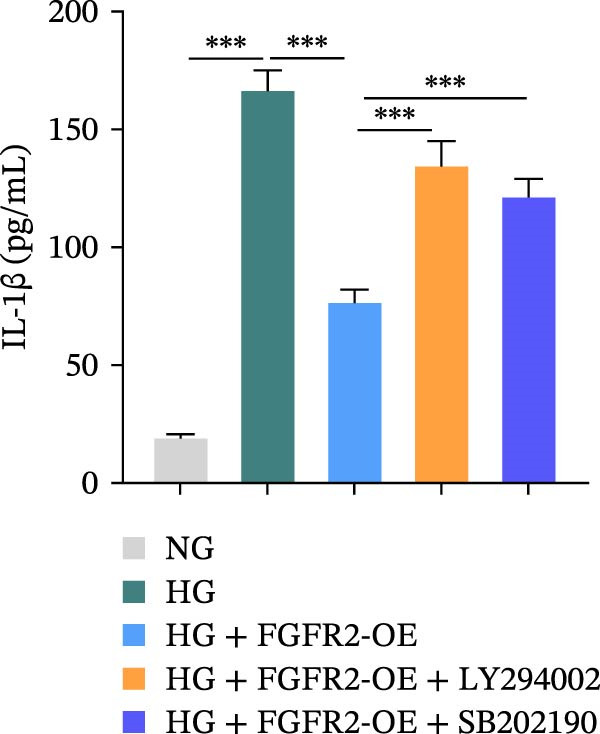
(H)

**Figure:**
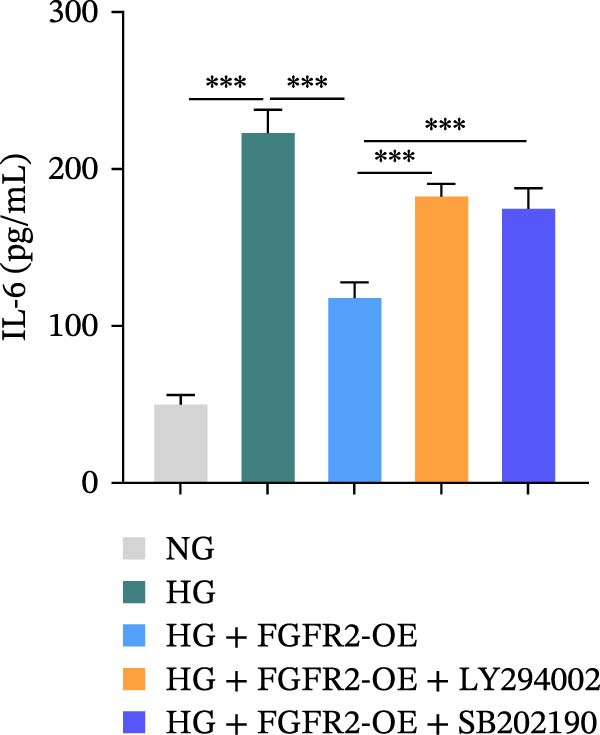
(I)

**Figure:**
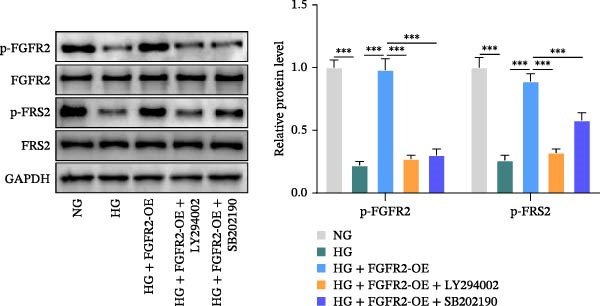
(J)

### 3.8. FGFR2 Loss‐of‐Function Exacerbates HG–Induced Injury Through Modulating Both PI3K/Akt and p38 MAPK Pathways

Next, we performed loss‑of‑unction experiments. HaCaT cells were transfected with FGFR2‑specific siRNA (si‑FGFR2), and knockdown efficiency was confirmed at both mRNA and protein levels (Supporting Information 3: Figure S2A,B). Compared to the HG + si‑NC group, the HG + si‑FGFR2 group showed a more pronounced reduction in FGFR2 mRNA and protein levels (Supporting Information 3: Figure S2C,D). Functional assays revealed that FGFR2 knockdown further exacerbated the HG‑induced decrease in cell viability (Supporting Information 3: Figure S2E) and migratory capacity (Supporting Information 3: Figure S2F,G), while promoting apoptosis (Supporting Information 3: Figure S2H,I) and the secretion of inflammatory cytokines (Supporting Information 3: Figure S2J–L). Mechanistically, FGFR2 knockdown led to a more profound suppression of the protein levels of p‑PI3K, p‑Akt, p‑mTOR, p‑FGFR2, p‐p38, and p‑FRS2 in HG‑stimulated HaCaT cells (Supporting Information 4: Figure S3A,B). Treatment with the PI3K activator 740Y‑P or the p38 MAPK activator anisomycin reversed the inhibition of PI3K/Akt/mTOR and p38 MAPK signaling induced by FGFR2 knockdown (Supporting Information 4: Figure S3C,D). Importantly, rescue experiments demonstrated that both activators effectively mitigated the damage caused by FGFR2 knockdown, including the restoration of cell viability, improved migration, reduced apoptosis, decreased inflammatory cytokine secretion, and partial reversal of the downregulation of p‑FGFR2 and p‑FRS2 (Supporting Information 5: Figure S4A–G). These findings further indicated that FGFR2 exerted its protective role against HG‑induced injury in HaCaT cells by coordinately activating both the PI3K/Akt and p38 MAPK signaling pathways.

## 4. Discussion

DFU is a serious complication of diabetes, and its causes involve multiple factors, mainly including neuropathy, abnormal microvascular blood circulation, secondary infection caused by foot trauma, and so on [21, 22]. In this study, 108 DE‐IRGs were identified in DFU and DFS tissues, which were mainly involved in cell chemotaxis, myeloid leukocyte migration, and leukocyte migration regulation. This suggests that the occurrence and development of DFU are closely related to the abnormal recruitment and migration of immune cells. These DE‐IRGs were also significantly enriched in the PI3K/Akt and MAPK signaling pathways. These pathways not only regulate cell proliferation, migration, and apoptosis but also are closely related to inflammatory responses and immune cell functions and may be important molecular mechanisms for the difficult healing of DFU [23, 24].

FGFR2 is a key member of the FGFR family and plays a crucial role in the cellular processes necessary for wound healing [14, 22, 25, 26]. Catechu extracts can be potentially applied to treat chronic diabetic wounds by upregulating the expression of FGFR2 and FGFR3 and activating NF‐kB and STAT3 pathways [27]. In this study, five core genes were ultimately screened out: *FGF2*, *FGF7*, *FGFR2*, *IGF1*, and *KITLG*, and FGFR2 demonstrated high diagnostic efficacy (AUC > 0.9). This suggests that FGFR2 might be a novel candidate biomarker for DFU. To clarify the specific function of FGFR2 in DFU, this study used HG–induced HaCaT cells as the in vitro model. Keratinocytes, as the main functional cells of the epidermis of the skin, have impaired proliferation and migration abilities, which is the core link of delayed wound healing in DFU [28, 29]. HG significantly reduced the mRNA and protein expression of FGFR2 in HaCaT cells. Overexpression of FGFR2 could reverse the decreased cell viability and migration ability induced by HG and simultaneously reduced cell apoptosis and the secretion of pro‐inflammatory factors. These findings indicate that FGFR2 has a protective effect in maintaining keratinocyte function, inhibiting inflammation and promoting tissue repair, providing experimental evidence for its characteristics as a therapeutic target.

Functional enrichment analysis indicated that FGFR2‐related targets were enriched in the PI3K/Akt pathway. Importantly, PI3K/Akt pathway plays a positive role in wound healing in diabetes [30, 31]. In this study, we found that HG stimulation inhibited the protein levels of p‐PI3K, p‐AKT, and p‐mTOR in HaCaT cells, while overexpression of FGFR2 could counteracted the effects of HG. In addition, PI3K‐specific inhibitor LY294002 could partially reverse the effect of FGFR2 overexpression on HG–induced HaCaT cells. This indicates that FGFR2 may be involved in the pathogenesis of DFU by regulating the PI3K/Akt/mTOR signaling. Dysregulation of the immune microenvironment is a key factor in the occurrence and development of DFU [32, 33]. Owing to the limited sample size of the GSE80178 dataset, the confidence of the deconvolution results from the CIBERSORT algorithm was low, and no reliable differential infiltration pattern of immune cell subsets was obtained after FDR correction. To address this limitation, we employed the EPIC algorithm for orthogonal validation. The analysis revealed that compared with DFS tissues, DFU tissues exhibited increased infiltration of B cells and CD8 + T cells, while CAFs infiltration was reduced, which is consistent with the previous reports [34]. Notably, Spearman correlation analysis confirmed that FGFR2 expression was negatively correlated with the infiltration levels of B cells and CD8 + T cells. This finding suggests that FGFR2 may attenuate the local inflammatory response in DFU by suppressing excessive infiltration of B cells and CD8 + T cells, thereby fostering a favorable immune microenvironment for wound healing. The present study did not explain the molecular mechanism by which FGFR2 modulates the infiltration of immune cells in DFU microenvironment. However, some previous studies have implied the significance and potential mechanism of FGFR2 in this process. FGFR2 signaling may induce the secretion of cytokines such as IL‐6 and IL‐8 through pathways such as STAT3 and NF‐κB and establishes an immunosuppressive microenvironment [14, 27, 35–38]. Some previous studies also report that it may induces PD‐L1 upregulation [37, 38]. At the level of matrix remodeling, it upregulates the expression and degradation of extracellular matrix components such as matrix metalloproteinase and collagen, and promotes the secretion of angiogenic factors, thereby altering the physical structure and nutrient supply of the tissue. These mechanisms interweave, making FGFR2 a potential regulatory node that coordinates the physical, chemical, immune, and metabolic dimensions of the microenvironment.

Reverse drug screening was conducted on 108 DE‐IRGs using the L1000FWD database, and 10 candidate drugs that could stably bind to the FGFR2 protein were obtained. Reversine is a 2,6‐disubstituted purine that can inhibit the kinase activity involved in cell cycle regulation and cytokine division, and has potential roles in anticancer treatment [39, 40]. As a classic PI3K inhibitor, LY294002 not only serves as a tool drug for verifying the PI3K/Akt pathway in this study but also demonstrates a high binding energy with FGFR2, suggesting that it may exert a dual regulatory effect by directly acting on FGFR2. This provides a new approach for the treatment of DFU. Neratinib is an FDA–approved drug targeting HER2/EGFR dual‐kinase and is an effective MST1 inhibitor that can protect pancreatic β cells in diabetic patients [41, 42]. However, its specific mechanism of action in DFU has not yet been clarified. QL‐XI‐92 and BRD‐K67414432, as relatively new compounds, their specific pharmacological effects and in vivo efficacy in DFU models still need to be further verified.

## 5. Conclusion

FGFR2 is a candidate immune‐associated marker of DFU. It promotes HG–induced proliferation, migration of HaCaT cells by activating the PI3K/Akt pathway, and inhibits apoptosis and inflammatory responses. These findings not only deepen the understanding of the molecular mechanism of DFU but also provide important references for the future development of diagnostic tools and therapeutic strategies targeting FGFR2. However, the in vitro model is difficult to fully mimic the complicated pathological microenvironment in DFU pathogenesis, lack of protein‐level validation in DFU tissues, and in vivo experimental confirmation, and the impact of FGFR2 on other skin cells (e.g., fibroblasts and endothelial cells) has not been deeply explored. These issues need to be further improved in subsequent research.

## Author Contributions

Fengrui Lei conceived and designed the experiments. Hailan Chen and Hongfei Sang performed the experiments. Yi Shi, Jie Pan, and Chuang Zhang analyzed the data (Yi Shi contributed to bioinformatics analysis and statistical tests, Jie Pan contributed to immune infiltration analysis and ROC curve evaluation, and Chuang Zhang contributed to experimental data quantification and visualization). Hailan Chen, Hongfei Sang, and Fengrui Lei wrote the paper.

## Funding

No funding was received for this manuscript.

## Disclosure

All authors have read and approved the final manuscript.

## Ethics Statement

The data used in the study are publicly available and allows unlimited reuse through an open license. Therefore, no ethical approval or informed consent was required in this study.

## Conflicts of Interest

The authors declare no conflicts of interest.

## Supporting Information

Additional supporting information can be found online in the Supporting Information section.

## Supporting information


**Supporting Information 1** Table S1: Differential expression genes (DEGs) between DFU and DFS in GSE80178 screened based on adj. *p* and |log_2_ FC| > 1. Table S2: Differentially expressed immune related genes (DE‐IRGs). Table S3: CytoHubba output results of DE‐IRGs. Table S4: Sample‐level data of CIBERSORT immune infiltration analysis. Table S5: Multiple‐testing correction results for differential infiltration of 22 immune cell subsets. Table S6: Multiple‐testing correction results for the correlation between FGFR2 expression and immune cell infiltration.

**Supporting Information 2** Figure S1: Verification of FGFR2 overexpression function in HG–induced human umbilical vein endothelial cells (HUVECs). (A, B) The mRNA (A) and protein (B) expression levels of FGFR2 in HUVECs transfected with empty plasmid (vector) or FGFR2 overexpression plasmid (FGFR2‐OE) were detected by RT‐qPCR and western blot, respectively. HUVECs were divided into four groups: normal glucose (NG), high glucose (HG), HG + vector (empty plasmid transfection), and HG + FGFR2‐OE (FGFR2 overexpression plasmid transfection). (C, D) FGFR2 mRNA (C) and protein (D) expression levels in HUVECs detected by RT‐qPCR and western blot, respectively. (E) Cell viability detected by CCK‐8 assay. (F, G) Cell migration ability evaluated by transwell assay. Scale bar = 100 μm. (H, I) Apoptosis rate analyzed by flow cytometry. (L) The levels of TNF‐α (J), IL‐1β (K), and IL‐6 (L) detected by ELISA. Data are expressed as mean ± SD (*n* = 3 independent biological replicates).  ^∗∗^ *p* < 0.01;  ^∗∗∗^ *p* < 0.001.

**Supporting Information 3** Figure S2: Effects of FGFR2 knockdown on HG–induced HaCaT cell injury. (A, B) FGFR2 knockdown efficiency of three specific siRNAs (si‐FGFR2#1, #2, #3) and negative control (si‐NC) in HaCaT cells, verified by RT‐qPCR (A) and western blot (B). (C, D) FGFR2 mRNA (C) and protein (D) expression levels in HaCaT cells from NG, HG, HG + si‐NC, and HG + si‐FGFR2#1 groups, detected by RT‐qPCR and western blot, respectively. (E) Cell viability of HG–induced HaCaT cells with FGFR2 knockdown, detected by CCK‐8 assay. (F, G) Cell migration ability of HG–induced HaCaT cells with FGFR2 knockdown, evaluated by transwell assay. Scale bar = 100 μm. (H, I) Apoptosis rate of HG–induced HaCaT cells with FGFR2 knockdown, analyzed by flow cytometry. (J–L) The levels of TNF‐α (J), IL‐1β (K), and IL‐6 (L) in HG–induced HaCaT cells with FGFR2 knockdown, detected by ELISA. Data are expressed as mean ± SD (*n* = 3 independent biological replicates).  ^∗∗^ *p* < 0.01;  ^∗∗∗^ *p* < 0.001.

**Supporting Information 4** Figure S3: Effects of FGFR2 knockdown on PI3K/Akt and p38 MAPK pathways in HG–induced HaCaT cells. (A, B) Protein expression levels of p‐PI3K, p‐Akt, p‐mTOR, p‐p38, p‐FGFR2, and p‐FRS2 in HaCaT cells from NG, HG, HG + si‐NC, and HG + si‐FGFR2#1 groups, detected by western blot. (C) Protein expression levels of p‐PI3K, p‐Akt, and p‐mTOR in HaCaT cells from NG, HG, HG + si‐FGFR2#1, and HG + si‐FGFR2#1+740Y‐P (PI3K activator) groups, detected by western blot. (D) Protein expression level of p‐p38 in HaCaT cells from NG, HG, HG + si‐FGFR2#1, and HG + si‐FGFR2#1 + anisomycin (p38 MAPK activator) groups, detected by western blot. Data are expressed as mean ± SD (*n* = 3 independent biological replicates).  ^∗^^∗^^∗^ *p* < 0.001.

**Supporting Information 5** Figure S4: Rescue effects of PI3K activator 740Y‐P and p38 MAPK activator anisomycin on FGFR2 knockdown‐induced HaCaT cell injury. HaCaT cells were divided into five groups: normal glucose (NG), high glucose (HG), HG + si‐FGFR2#1 (FGFR2 knockdown), HG + si‐FGFR2#1+740Y‐P (PI3K activator), and HG + si‐FGFR2#1 + anisomycin (p38 MAPK activator). (A) Cell viability detected by CCK‐8 assay. (B) Cell migration ability evaluated by transwell assay. Scale bar = 100 μm. (C) Apoptosis rate analyzed by flow cytometry. (F) The levels of TNF‐α (D), IL‐1β (E), and IL‐6 (F) detected by ELISA. (G) Protein expression levels of p‐FGFR2 and p‐FRS2 detected by western blot. Data are expressed as mean ± SD (*n* = 3 independent biological replicates).  ^∗^^∗^ *p* < 0.01;  ^∗^^∗^^∗^ *p* < 0.001.

## Data Availability

The datasets used in this study are available in the GEO repository: GSE80178 (https://www.ncbi.nlm.nih.gov/geo/query/acc.cgi?acc=GSE80178), GSE199939 (https://www.ncbi.nlm.nih.gov/geo/query/acc.cgi?acc=GSE199939), and GSE134431 (https://www.ncbi.nlm.nih.gov/geo/query/acc.cgi?acc=GSE134431). The lists of differentially expressed genes (DEGs) and differentially expressed immune‐related genes (DE‐IRGs; including gene symbols, log_2_ fold change, adjusted *p*‐values, and expression trends) are provided in Supporting Information 1: Tables S1 and S2, respectively. The output results of cytoHubba (including the top 15 genes ranked by each of the nine topological algorithms and their corresponding scores) are available in Supporting Information 1: Table S3. To address the concerns about CIBERSORT immune infiltration analysis, we have supplemented the following data tables: Supporting Information 1: Table S4 (sample‐level *p*‐values of CIBERSORT), Supporting Information 1: Table S5 (FDR–adjusted results for differential infiltration of 22 immune cell subsets), Supporting Information 1: Table S6 (FDR–adjusted results for FGFR2–immune cell correlation). All other data generated or analyzed during this study are included in this published article and its supporting information files. Further details are available from the corresponding author upon reasonable request.
