# Supplementary figures and images for: FGFR2 is a Candidate Immune‐Associated Marker of Diabetic Foot Ulcer That Promotes Keratinocyte Function by Activating the PI3K/Akt and MAPK Pathways
Source: Mediators Inflamm. 2026 Apr 15;2026:3260549. doi: 10.1155/mi/3260549 (PMC13081191; doi:10.1155/mi/3260549)

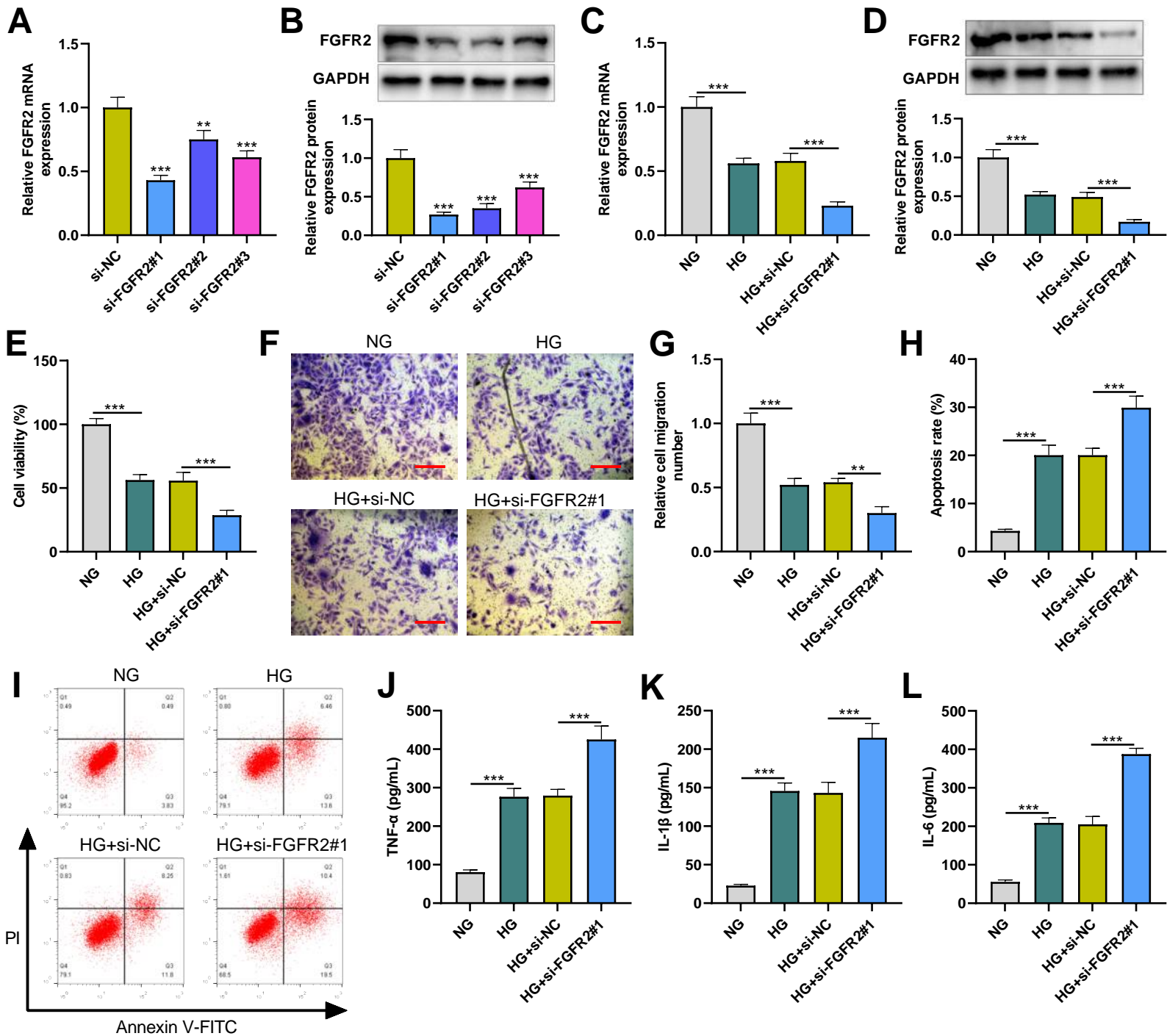

Supplement: Supplementary file 2 — Supporting Information 2 Figure S1: Verification of FGFR2 overexpression function in HG–induced human umbilical vein endothelial cells (HUVECs). (A, B) The mRNA (A) and protein (B) expression levels of FGFR2 in HUVECs transfected with empty plasmid (vector) or FGFR2 overexpression plasmid (FGFR2‐OE) were detected by RT‐qPCR and western blot, respectively. HUVECs were divided into four groups: normal glucose (NG), high glucose (HG), HG + vector (empty plasmid transfection), and HG + FGFR2‐OE (FGFR2 overexpression plasmid transfection). (C, D) FGFR2 mRNA (C) and protein (D) expression levels in HUVECs detected by RT‐qPCR and western blot, respectively. (E) Cell viability detected by CCK‐8 assay. (F, G) Cell migration ability evaluated by transwell assay. Scale bar = 100 μm. (H, I) Apoptosis rate analyzed by flow cytometry. (L) The levels of TNF‐α (J), IL‐1β (K), and IL‐6 (L) detected by ELISA. Data are expressed as mean ± SD (n = 3 independent biological replicates). ∗∗ p < 0.01; ∗∗∗ p < 0.001. [file MI-2026-3260549-s002.pdf]

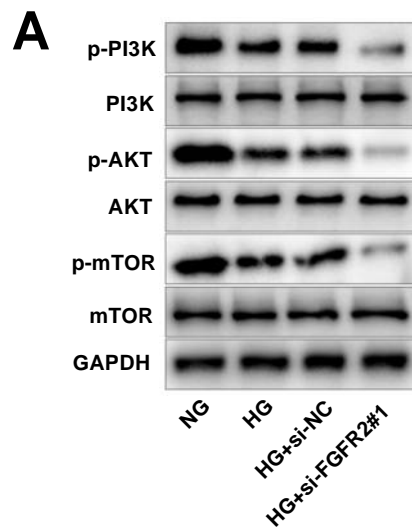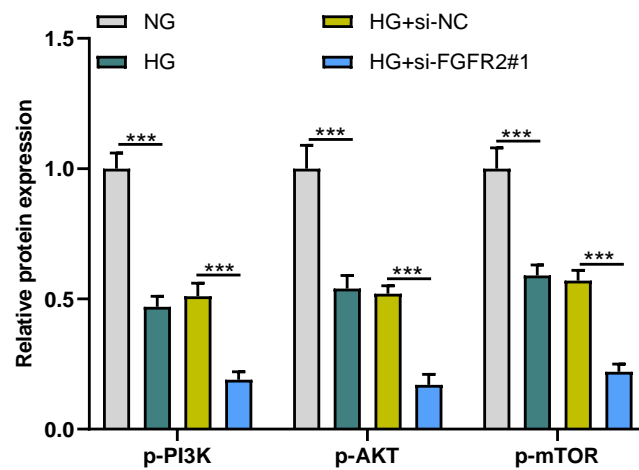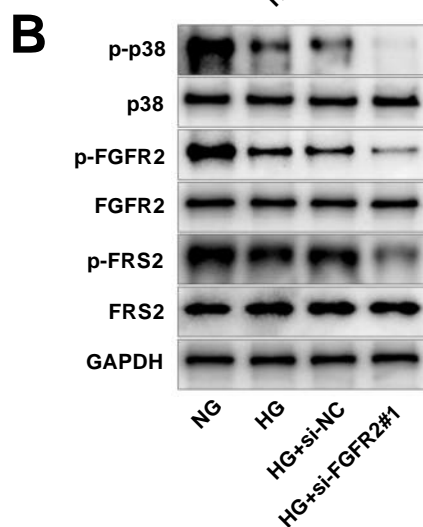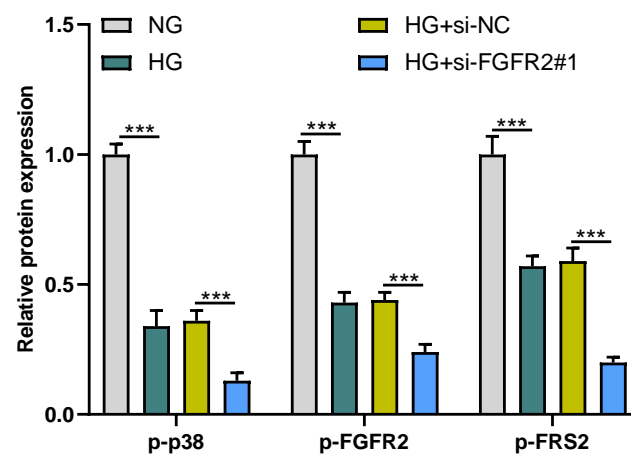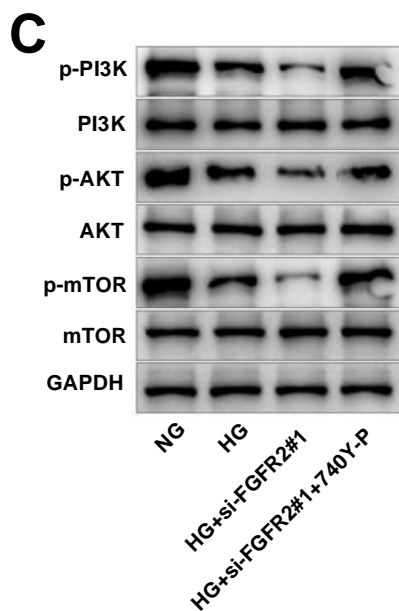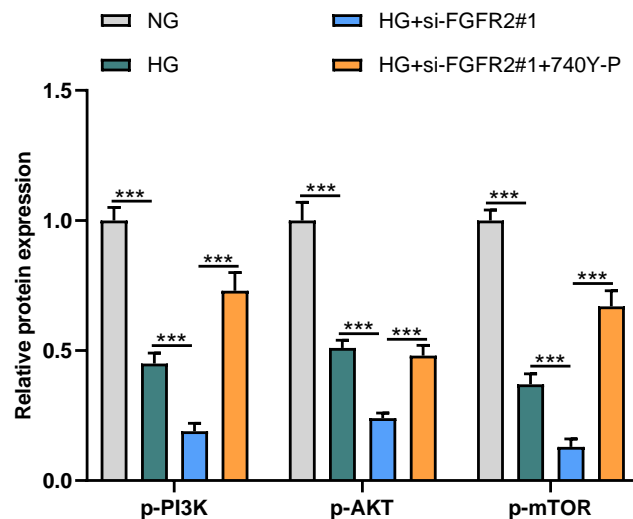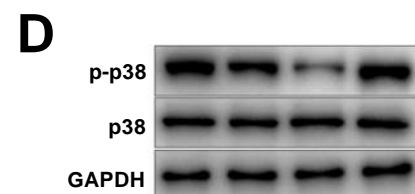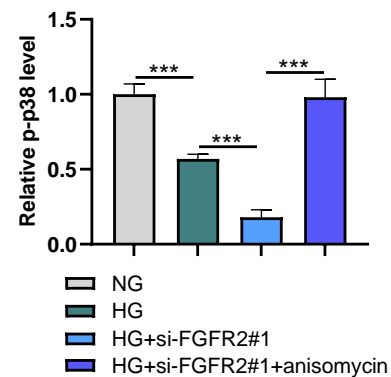

Supplement: Supplementary file 3 — Supporting Information 3 Figure S2: Effects of FGFR2 knockdown on HG–induced HaCaT cell injury. (A, B) FGFR2 knockdown efficiency of three specific siRNAs (si‐FGFR2#1, #2, #3) and negative control (si‐NC) in HaCaT cells, verified by RT‐qPCR (A) and western blot (B). (C, D) FGFR2 mRNA (C) and protein (D) expression levels in HaCaT cells from NG, HG, HG + si‐NC, and HG + si‐FGFR2#1 groups, detected by RT‐qPCR and western blot, respectively. (E) Cell viability of HG–induced HaCaT cells with FGFR2 knockdown, detected by CCK‐8 assay. (F, G) Cell migration ability of HG–induced HaCaT cells with FGFR2 knockdown, evaluated by transwell assay. Scale bar = 100 μm. (H, I) Apoptosis rate of HG–induced HaCaT cells with FGFR2 knockdown, analyzed by flow cytometry. (J–L) The levels of TNF‐α (J), IL‐1β (K), and IL‐6 (L) in HG–induced HaCaT cells with FGFR2 knockdown, detected by ELISA. Data are expressed as mean ± SD (n = 3 independent biological replicates). ∗∗ p < 0.01; ∗∗∗ p < 0.001. [file MI-2026-3260549-s005.pdf]

**A**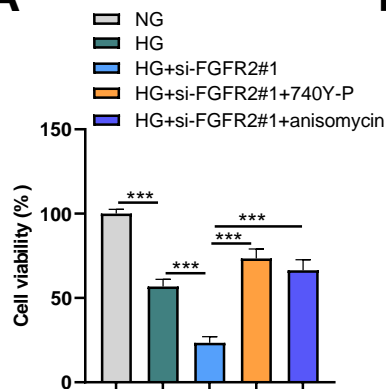**B**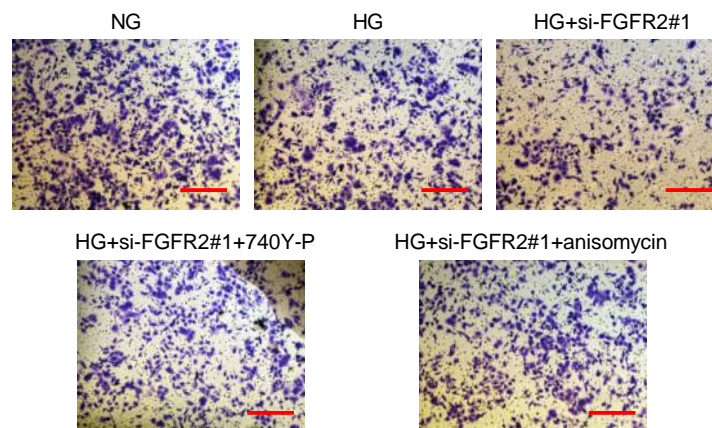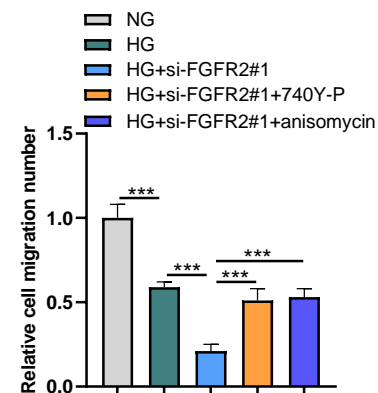**C**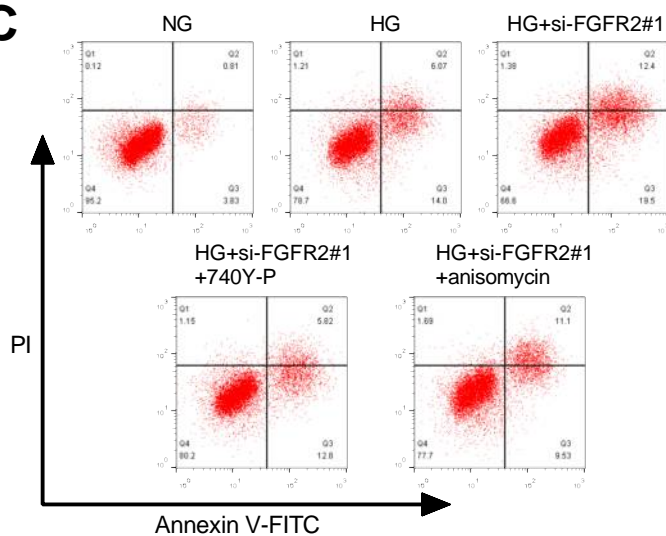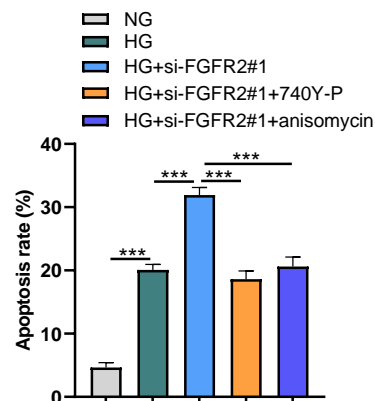**D**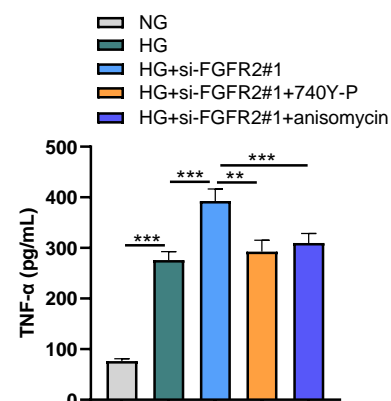**E**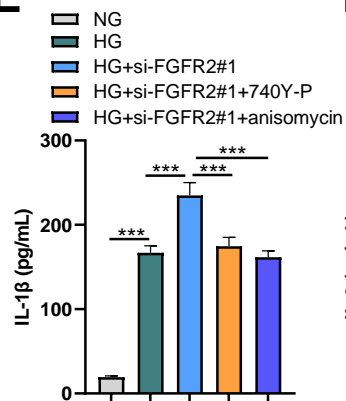**F**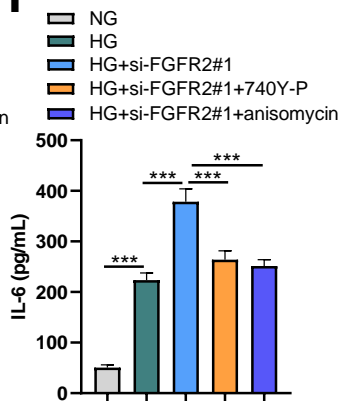**G**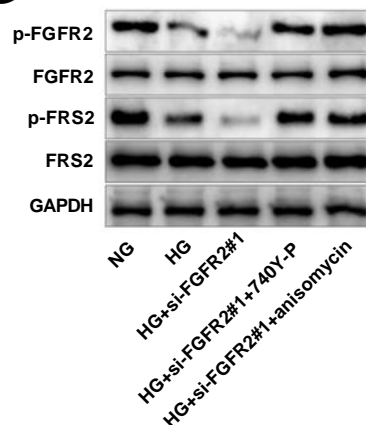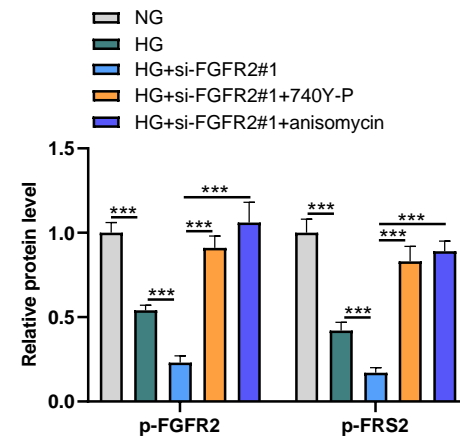

Supplement: Supplementary file 4 — Supporting Information 4 Figure S3: Effects of FGFR2 knockdown on PI3K/Akt and p38 MAPK pathways in HG–induced HaCaT cells. (A, B) Protein expression levels of p‐PI3K, p‐Akt, p‐mTOR, p‐p38, p‐FGFR2, and p‐FRS2 in HaCaT cells from NG, HG, HG + si‐NC, and HG + si‐FGFR2#1 groups, detected by western blot. (C) Protein expression levels of p‐PI3K, p‐Akt, and p‐mTOR in HaCaT cells from NG, HG, HG + si‐FGFR2#1, and HG + si‐FGFR2#1+740Y‐P (PI3K activator) groups, detected by western blot. (D) Protein expression level of p‐p38 in HaCaT cells from NG, HG, HG + si‐FGFR2#1, and HG + si‐FGFR2#1 + anisomycin (p38 MAPK activator) groups, detected by western blot. Data are expressed as mean ± SD (n = 3 independent biological replicates). ∗ ∗ ∗ p < 0.001. [file MI-2026-3260549-s004.pdf]

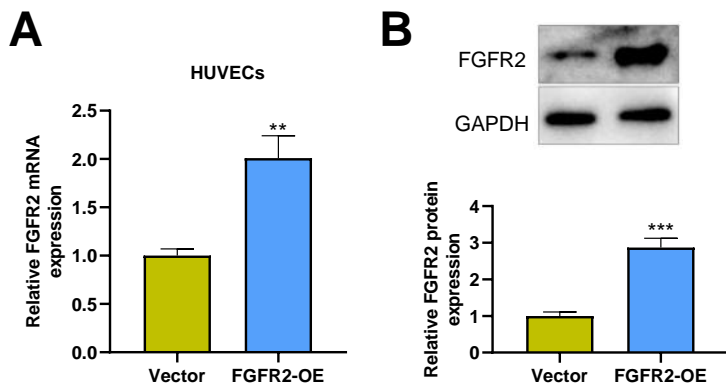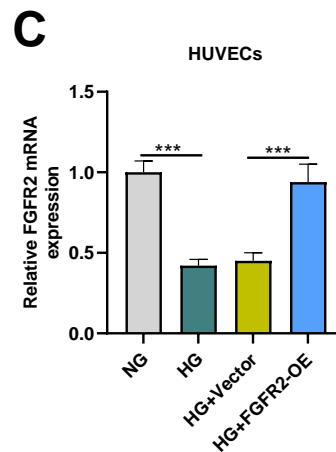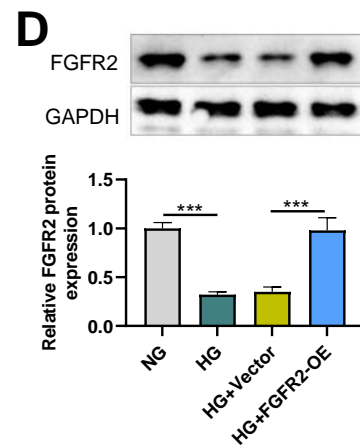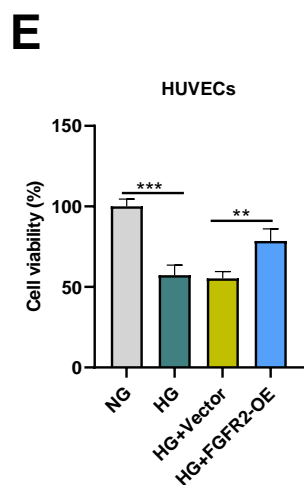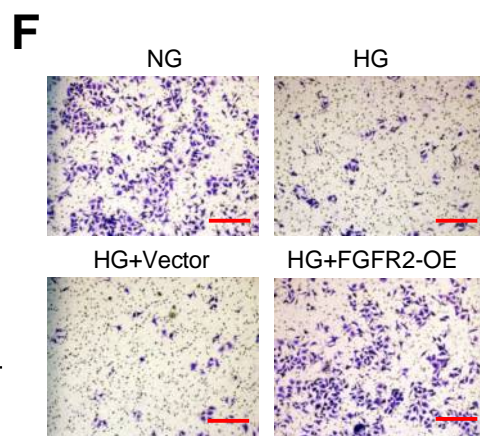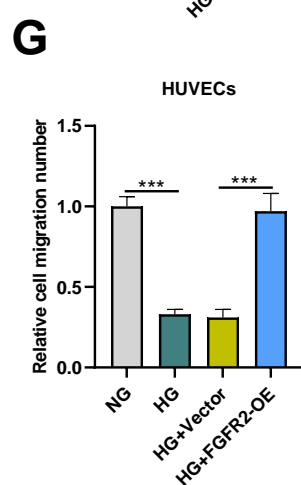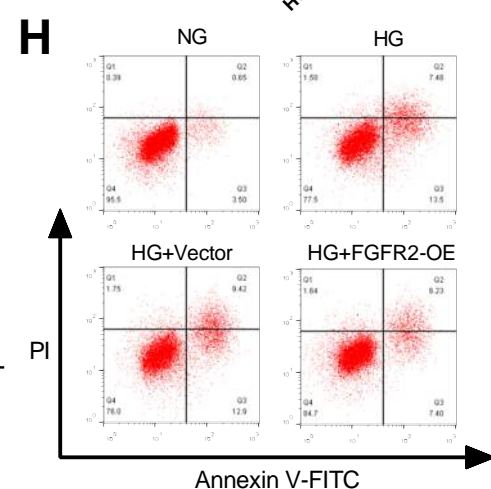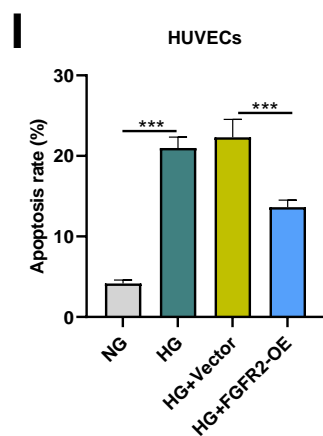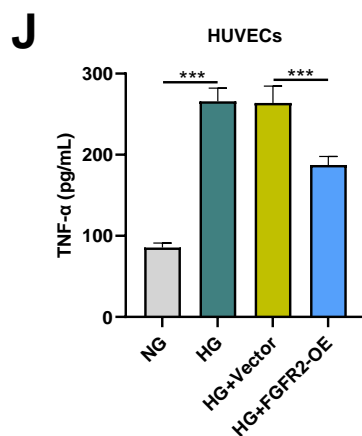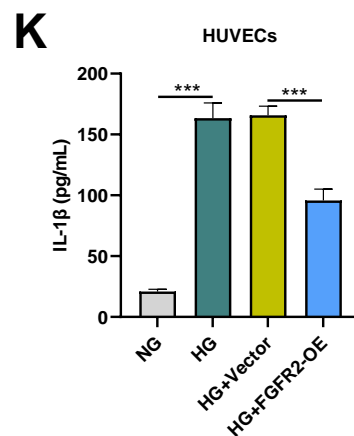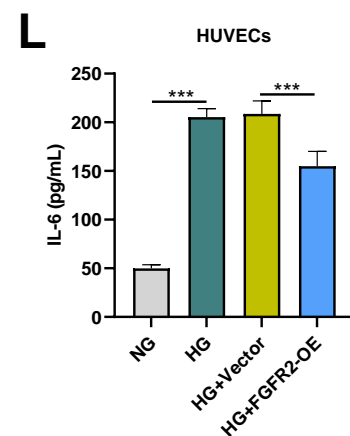

Supplement: Supplementary file 5 — Supporting Information 5 Figure S4: Rescue effects of PI3K activator 740Y‐P and p38 MAPK activator anisomycin on FGFR2 knockdown‐induced HaCaT cell injury. HaCaT cells were divided into five groups: normal glucose (NG), high glucose (HG), HG + si‐FGFR2#1 (FGFR2 knockdown), HG + si‐FGFR2#1+740Y‐P (PI3K activator), and HG + si‐FGFR2#1 + anisomycin (p38 MAPK activator). (A) Cell viability detected by CCK‐8 assay. (B) Cell migration ability evaluated by transwell assay. Scale bar = 100 μm. (C) Apoptosis rate analyzed by flow cytometry. (F) The levels of TNF‐α (D), IL‐1β (E), and IL‐6 (F) detected by ELISA. (G) Protein expression levels of p‐FGFR2 and p‐FRS2 detected by western blot. Data are expressed as mean ± SD (n = 3 independent biological replicates). ∗ ∗ p < 0.01; ∗ ∗ ∗ p < 0.001. [file MI-2026-3260549-s003.pdf]
